# A Large Genetic Causal Analysis of the Gut Microbiota and Urological Cancers: A Bidirectional Mendelian Randomization Study

**DOI:** 10.3390/nu15184086

**Published:** 2023-09-21

**Authors:** Zhaofa Yin, Bohan Liu, Shijian Feng, Yushi He, Cai Tang, Pengan Chen, Xinyi Wang, Kunjie Wang

**Affiliations:** Department of Urology, Institute of Urology (Laboratory of Reconstructive Urology), National Clinical Research Center for Geriatrics, West China Hospital, Sichuan University, No. 37 Guo Xue Xiang, Chengdu 610041, China; ldszxyyyzf@163.com (Z.Y.); fengshijian@wchscu.cn (S.F.); 13348939543@163.com (Y.H.); tc1989@163.com (C.T.); cpa_scu@163.com (P.C.);

**Keywords:** gut microbiota, urological cancer, mendelian randomization, genetics

## Abstract

Background: Several observational studies and clinical trials have shown that the gut microbiota is associated with urological cancers. However, the causal relationship between gut microbiota and urological cancers remains to be elucidated due to many confounding factors. Methods: In this study, we used two thresholds to identify gut microbiota GWAS from the MiBioGen consortium and obtained data for five urological cancers from the UK biobank and Finngen consortium, respectively. We then performed a two-sample Mendelian randomization (MR) analysis with Wald ratio or inverse variance weighted as the main method. We also performed comprehensive sensitivity analyses to verify the robustness of the results. In addition, we performed a reverse MR analysis to examine the direction of causality. Results: Our study found that family *Rikenellaceae*, genus *Allisonella*, genus *Lachnospiraceae UCG001*, genus *Oscillibacter*, genus *Eubacterium coprostanoligenes group*, genus *Eubacterium ruminantium group*, genus *Ruminococcaceae UCG013*, and genus *Senegalimassilia* were related to bladder cancer; genus *Ruminococcus torques group*, genus *Oscillibacter*, genus *Barnesiella*, genus *Butyricicoccus*, and genus *Ruminococcaceae UCG005* were related to prostate cancer; class *Alphaproteobacteria*, class *Bacilli*, family *Family XI*, genus *Coprococcus2*, genus *Intestinimonas*, genus *Lachnoclostridium*, genus *Lactococcus*, genus *Ruminococcus torques group*, and genus *Eubacterium brachy group* were related to renal cell cancer; family *Clostridiaceae 1*, family *Christensenellaceae*, genus *Eubacterium coprostanoligenes group*, genus *Clostridium sensu stricto 1*, and genus *Eubacterium eligens group* were related to renal pelvis cancer; family *Peptostreptococcaceae*, genus *Romboutsia*, and genus *Subdoligranulum* were related to testicular cancer. Comprehensive sensitivity analyses proved that our results were reliable. Conclusions: Our study confirms the role of specific gut microbial taxa on urological cancers, explores the mechanism of gut microbiota on urological cancers from a macroscopic level, provides potential targets for the screening and treatment of urological cancers, and is dedicated to providing new ideas for clinical research.

## 1. Introduction

With the proliferation of the populace and the escalation of societal senescence, the incidence and prevalence of cancer have dramatically grown [1]. The data showed that urological cancers accounted for 13.1% of new cancer cases and 7.9% of total cancer mortality [2]. The prevalent urological cancers are bladder cancer, prostate cancer, renal cell cancer, renal pelvis cancer, and testicular cancer [3]. Compared with 1990, the number of patients with urological cancers has increased by 2.5 fold, and the number of deaths has increased by 1.6 fold [4]. According to statistics, in the United States, there were 17,100 additional deaths from bladder cancer, 34,500 from prostate cancer, and 13,920 from renal cell and renal pelvis cancer in 2022 [2]. Testicular cancer remains relatively rare, accounting for 0.4% of new cancer cases [5]. Compared to other cancers, urological cancers develop slowly and can easily be cured by early detection and treatment [6]. However, the early symptoms of urological cancers are not obvious, and cystoscopy, tissue biopsy, and imaging examinations limit their use in large-scale screening due to invasiveness, cost, and ionizing radiation [7]. Therefore, it is very important to find new targets suitable for large-scale screening and prevention of urological cancers.

In recent years, with the discovery of the gut–kidney axis, gut–prostate axis, and gut–testis axis, the association between gut microbiota and urological cancers has gained significant attention [8,9,10]. The gut microbiota refers to the vast community of microorganisms residing in the gastrointestinal tract, primarily composed of bacteria, fungi, viruses, and other microbial species, 98% of which are bacteria [11]. The gut microbiota is a mutualistic symbiosis in humans that plays a crucial role in maintaining human health and has a profound impact on various disease processes, including urological cancers [12,13,14]. To date, several observational studies have shown differences between healthy individuals and patients with urological cancers in the composition and diversity of the gut microbiota. In a Chinese case–control study, He et al. observed a lower abundance of *Prevotella* in the intestines of bladder cancer patients [15]. In an observational study in the United States, Liss et al. found that *Bacteroides* and *Streptococci* were more abundant in the intestines of prostate cancer patients [16]. Several animal model experiments have also reported the involvement of the gut microbiota in the progression of urological cancers via intricate signaling pathways [13,17]. 

The association between gut microbiota and urological cancers is a rapidly evolving field of research. It holds great promise for the development of novel diagnostic, preventive, and therapeutic strategies. Understanding the intricate relationships between the gut microbiota and urological cancers could develop the microbiota-based therapies such as microbiota transplantation, probiotic therapy, and dietary therapy, as well as novel targets for urologic cancer screening [18]. Clinicians can help identify individuals at higher risk based on their gut microbiota profiles and guide personalized interventions to mitigate that risk, making personalized treatment for urologic cancers possible.

Unfortunately, the conclusions of most current observational studies predominantly rely on the analysis of the composition and changes in gut microbiota in patients’ feces. Traditional observational studies are limited by inherent flaws, including environmental confounders, selection bias, and reverse causation [19]. The establishment of models in animal experiments focuses on transplanting gut microbiota into mice and deriving results, and the gut microbiota of these mice is susceptible to various influences, including factors such as dietary patterns and antibiotic usage [20]. In conclusion, the relationship between gut microbiota and urological cancers remains to be elucidated. Although randomized controlled trials (RCTs) are the gold standard for verifying causality, the extremely large number of gut microbial species and the long latency period from gut microbiota imbalance to cancer development make RCT difficult to apply in a real clinical setting [21]. Therefore, a new approach is needed to explore the causal relationship between gut microbiota and urological cancers.

We use Mendelian randomization (MR) to explore the causal relationship between the gut microbiota and urological cancers. In MR analysis, genetic variations are utilized as instrumental variables (IVs) to explore the causal association between exposure and outcome [22]. Since genetic variants originate from parents and are randomly assigned at conception, properly conducted MR analysis can prevent reverse causality and lessen bias caused by environmental variables [23].

In this study, we performed a bidirectional two-sample MR analysis to explore the causal relationship between gut microbiota and five urological cancers, including bladder cancer, prostate cancer, renal cell cancer, renal pelvis cancer, and testicular cancer. Based on the results of the MR analysis, we tried to elucidate the role of the gut microbiota in urological cancers, find new targets for cancer screening and prevention, and pave the way for the development of microbiota-based interventions, such as microbiota transplantation, probiotic therapy, and dietary therapy [18].

## 2. Materials and Methods

### 2.1. Study Design

**Figure 1:**
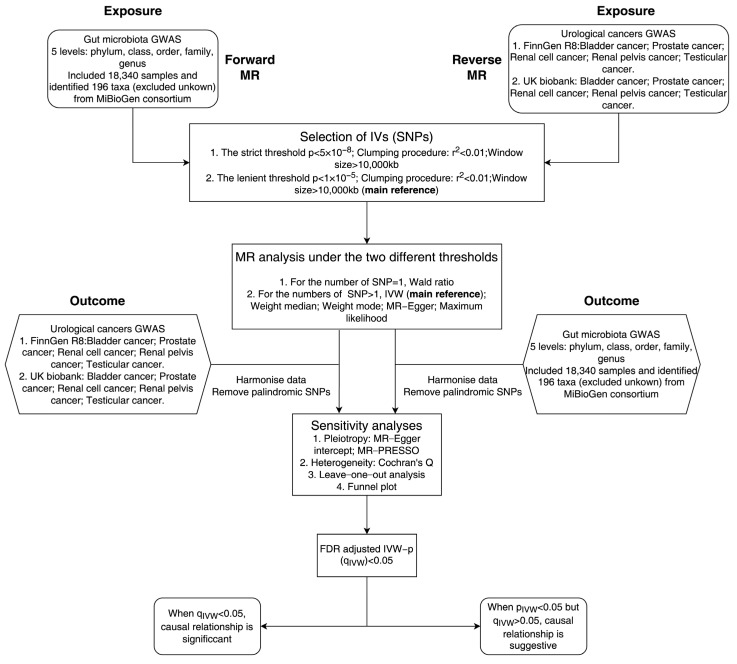
Study design and flowchart of MR analysis in this study.

Gut microbiota was defined as exposure and five urological cancers as the outcome (reverse MR analysis: urological cancer as exposure and gut microbiota as outcome). The IVs were screened out with a series of quality control procedures and analyzed using MR (Figure 1). Our MR analysis was based on three assumptions: (1) IVs were significantly associated with exposure; (2) IVs were not associated with any confounding factors; (3) IVs did not affect outcomes in any way other than exposure [24].

### 2.2. Data Sources

Single-nucleotide polymorphisms (SNPs) associated with the gut microbiota were obtained from the largest genome-wide association study (GWAS) summary data to date published by the MiBioGen consortium [25]. This is a large-scale multi-ethnic GWAS involving 18,340 participants in 24 cohorts, 72.3% of whom had European ancestry (*n* = 13,266). This study analyzed the microbial composition and classified microbiota using direct taxonomic binning by targeting three distinct variable regions (V4, V3–V4, V1–V2) of 16S ribosomal RNA in participants. Genetic variants in microbiota hosts were identified using microbiota quantitative trait loci mapping analysis. Kurilshikov A et al. also adjusted for sex, age, technical covariates, and genetic principal components. GWAS finally included 211 taxa (131 genera, 35 families, 20 orders, 16 classes, and 9 phyla) [25].

We obtained GWAS summary data for five urological cancers from FinnGen Biobank R8 and UK biobank, respectively [26,27]. Five urological cancers were diagnosed according to ICD-O-3, controls excluding all cancers. More information on the above can be found in the Supplementary Tables S1–S6.

### 2.3. Instrument Variable Selection

To ensure that the results of MR analysis are stable and reliable, we used the following criteria to screen IVs: (1) SNPs that were statistically significantly associated with the gut microbiome were chosen as IVs (*p* < 5 × 10^−8^). However, only a small amount of SNPs were chosen as IVs. To explore more comprehensive results, we set a more lenient threshold and used the filtered SNPs as the second IV set (*p* < 1 × 10^−5^). (2) To avoid linkage disequilibrium (LD), we selected the independent SNPs (r^2^ < 0.01 and distance > 10,000 kb) using the clumping procedure. (3) SNPs with minor allele frequencies (MAF) below 0.01 were excluded. (4) We removed duplicate SNPs and palindromic SNPs. (5) We searched Phenoscanner (http://www.phenoscanner.medschl.cam.ac.uk/, accessed on 28 April 2023), a database that can be used to find SNP-associated phenotypes, and removed SNPs directly related (*p* = 5 × 10^−8^) to five urological cancers [28]. (6) We calculated F-statistics (*F*), preventing the impact of weak IVs on the results, with the formula $F=\frac{R^{2}\;\times \;\left(N\;-\;1\;-\;K\right)}{\left(1\;-\;R^{2}\right)\times K}$, ($R^{2}=2\times {beta}^{2}\times MAF\times \left(1-MAF\right)$, *N* = sample size, *K* = number of IVs). *F* > 10 was generally considered a threshold for strong IVs [29]. Therefore, we excluded IVs with *F* < 10 when performing MR analysis.

### 2.4. MR Analysis

We performed MR analysis to explore the causal relationship between the gut microbiota and five urological cancers. For taxa with only one IV, we performed the Wald ratio for MR analysis [30]. For taxa with more than one IV, inverse variance-weighted (IVW) was the main statistical method for MR analysis. Through meta-analysis, the IVW integrated the impacts of individual IVs into a total weighted effect. Therefore, this method was reliable when all IVs were valid [31]. In addition, we used weighted median, maximum likelihood, weighted mode, and MR–Egger methods for complementary and alternative analyses. When more than 50% of IVs were valid, the results of the weighted-median method were reliable [32]. The maximum likelihood ratio measured the probability of a distribution parameter [33]. Based on the similarity of causality, the weighted mode method could divide SNPs into different subsets and assess the causal connection between exposure and outcome using the subset with the highest number of SNPs [34]. Horizontal pleiotropy could be calculated with MR egger. However, affected by the SNPs, the result may not be accurate [32]. When the results of the five MR analysis methods were different, we gave priority to the results of IVW.

To avoid increased Type 1 errors from multiple hypothesis testing, we corrected the significance threshold using the false discovery rate (FDR) correction [35]. Considering that the q-value (*q*) corrected with microbiota counts were too stringent, we used the number of species of the MR analysis method for correction. Corrected threshold *q* < 0.05 was considered significant. When *p* < 0.05 but *q* > 0.05, gut microbiota were considered associated with urological cancers.

### 2.5. Sensitivity Analyses

For taxa with more than 2 SNPs, we performed a series of sensitivity analyses. In the MR–Egger regression, intercepts were used to test directional horizontal pleiotropic effects [36]. We also used MR Pleiotropy RESidual Sum and Outlier (MR-PRESSO) global test for pleiotropy analysis and corrected estimates by removing outliers (outlier test) if necessary [37]. In addition, we used Cochran’s Q statistic and funnel plot to examine the heterogeneity of IVW and the MR–Egger regression methods. If there was heterogeneity in the results (*p* < 0.05), MR analysis was performed using the random effects model IVW. To determine if certain SNPs may affect on the results of the MR analysis, we used the leave-one-out test.

### 2.6. Reverse MR Analysis

To explore whether urological cancer has a causal effect on the important gut microbial taxa identified in the forward MR analysis, we performed a reverse MR analysis (five urological cancers as exposures and important gut microbial taxa as outcomes). The analysis process was consistent with the forward MR analysis. The MR Steiger directionality test was used to examine whether there was a directional causality between exposure and outcome [38].

The MR analyses were performed using “TwoSampleMR” (version 0.5.6), “MendelianRandomization” (version 0.6.0), “MRPRESSO” (version 1.0), and “qvalue” (version 1.0) packages in R (version 4.2.1). Statistical significance was assigned to a result with *p* < 0.05 (two-sided).

## 3. Results

### 3.1. SNPs Selection

After removing 15 unknown taxa, our study included 196 taxa (9 phyla, 16 classes, 20 orders, 33 families, and 119 genera) for MR analysis. According to the screening criteria of IVs, we selected 22 SNPs (one SNP for phylum, one SNP for class, three SNPs for order, five SNPs for family, and twelve SNPs for genus) at *p* < 5 × 10^−8^ level and 2238 SNPs (108 SNPs for phylum, 194 SNP for class, 237 SNPs for order, 414 SNPs for family, and 1306 SNPs for genus) at *p* < 1 × 10^−5^. The F-statistics for all SNPs were all > 10 (27.8–106.6 at *p* < 5 × 10^−8^, 10.2–106.6 at *p* < 1 × 10^−5^) (Supplementary Tables S1–S6). The results showed that all SNPs were effective strong IVs, and instrumental bias would not affect the results of MR analysis.

### 3.2. Forward MR Analysis

For the taxa with only one SNP, q=p. For the taxa with more than 1 SNP, $q=p\times \frac{m}{k}$. M represented the number of MR analysis methods. Sorting the *p*-values from smallest to largest, *k* represented the ranking of *p*-values.

### 3.3. Bladder Cancer

In MR analysis at the *p* < 5 × 10^−8^ level, we found that the genus *Allisonella* reduced the risk of bladder cancer (odds ratio (OR) = 0.55, 95% confidence interval (CI) = 0.37–0.82, *p* = 3.37 × 10^−3^, Wald ratio) and the genus *Eubacterium coprostanoligenes group* (OR = 4.27, 95% CI = 1.56–11.68, *p* = 4.72 × 10^−3^, Wald ratio) increased the risk of bladder cancer (Table 1).

**Figure 2:**
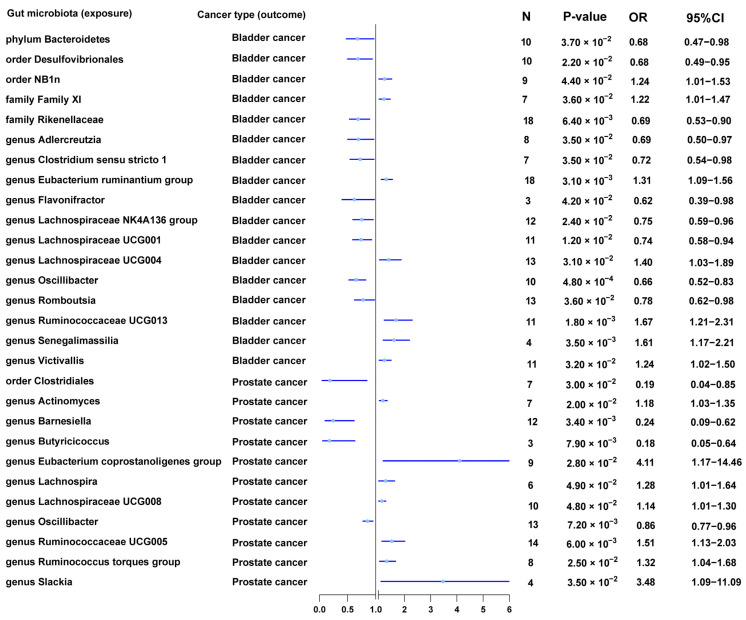
MR results of causal effects between gut microbiota and urological cancers (Bladder cancer and Prostate cancer) (*p* < 1 × 10^−5^).

In MR analysis at the *p* < 1 × 10^−5^ level, we found that family *Rikenellaceae* (OR = 0.69, 95% CI = 0.53–0.90, *q* = 1.59 × 10^−2^, IVW), genus *LachnospiraceaeUCG001* (OR = 0.74, 95% CI = 0.58–0.94, *q* = 2.66 × 10^−2^, IVW), and genus *Oscillibacter* (OR = 0.66, 95% CI = 0.52–0.83, *q* = 1.21 × 10^−3^, IVW) reduced the risk of bladder cancer; genus *Eubacterium ruminantium group* (OR = 1.31, 95% CI = 1.09–1.56, *q* = 1.53 × 10^−2^, IVW), genus *RuminococcaceaeUCG013* (OR = 1.67, 95% CI = 1.21–2.31, *q* = 8.76 × 10^−3^, IVW), and genus *Senegalimassilia* (OR = 1.61, 95% CI = 1.17–2.21, *q* = 1.32 × 10^−2^, IVW) increased the risk of bladder cancer (Table 2 and Figure 2).

### 3.4. Prostate Cancer

In MR analysis at the *p* < 5 × 10^−8^ level, we found that the genus *Ruminococcus torques group* (OR = 1.96, 95% CI = 1.18–3.25, *p* = 9.24 × 10^−3^, Wald ratio) increased the risk of prostate cancer (Table 1).

In MR analysis at the *p* < 1 × 10^−5^ level, we found that the genera *Oscillibacter* (OR = 0.86, 95% CI = 0.77–0.96, *q* = 2.42 × 10^−2^, IVW), *Barnesiella* (OR = 0.24, 95% CI = 0.09–0.62, *q* = 1.23 × 10^−2^, IVW), and *Butyricicoccus* (OR = 0.18, 95% CI = 0.05–0.64, *q* = 2.94 × 10^−2^, IVW) reduced the risk of prostate cancer; the genus *RuminococcaceaeUCG005* (OR = 1.51, 95% CI = 1.13–2.03, *q* = 3.02 × 10^−2^, IVW) increased the risk of prostate cancer (Table 2 and Figure 2).

### 3.5. Renal Cell Cancer

In MR analysis at the *p* < 5 × 10^−8^ level, we found that the genus *Ruminococcus torques group* (OR = 4.11, 95% CI = 1.34–12.67, *p* = 1.37 × 10^−2^, Wald ratio) increased the risk of renal cell cancer (Table 1).

**Figure 3:**
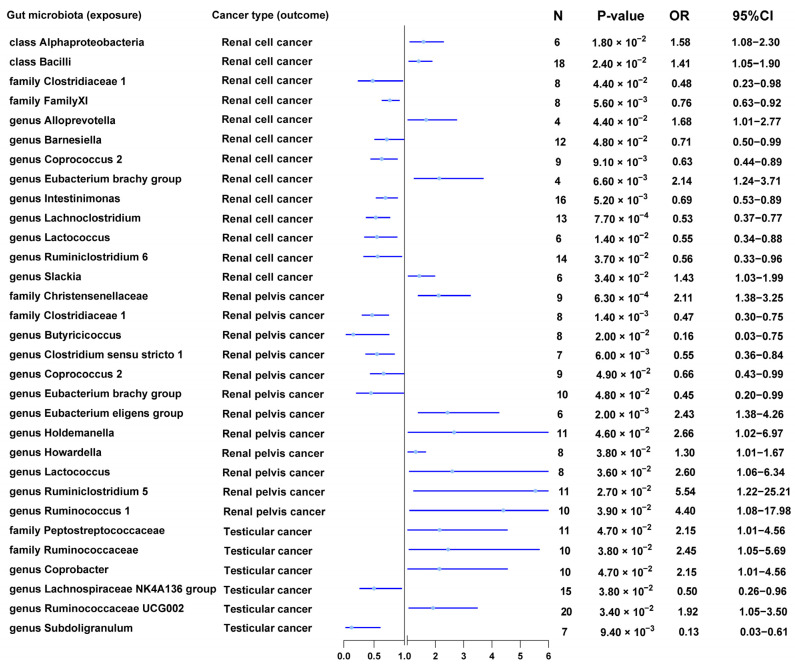
MR results of causal effects between gut microbiota and urological cancers (Renal cell cancer, Renal pelvis cancer, and Testicular cancer) (*p* < 1 × 10^−5^).

In MR analysis at the *p* < 1 × 10^−5^ level, we found that the family *Family XI* (OR = 0.76, 95% CI = 0.63–0.92, *q* = 1.80 × 10^−2^, IVW), the genera *Coprococcus 2* (OR = 0.63, 95% CI = 0.44–0.89, *q* = 2.44 × 10^−2^, IVW), *Intestinimonas* (OR = 0.69, 95% CI = 0.53–0.89, *q* = 1.89 × 10^−2^, IVW), *Lachnoclostridium* (OR = 0.53, 95% CI = 0.37–0.77, *q* = 1.92 × 10^−3^, IVW), and *Lactococcus* (OR = 0.55, 95% CI = 0.34–0.88, *q* = 3.64 × 10^−2^, IVW) reduced the risk of renal cell cancer; the classes *Alphaproteobacteria* (OR = 1.58, 95% CI = 1.08–2.30, *q* = 4.76 × 10^−2^, IVW) and *Bacilli* (OR = 1.41, 95% CI = 1.05–1.90, *q* = 4.22 × 10^−2^, IVW), and the genus *Eubacterium brachy group* (OR = 2.14, 95% CI = 1.24–3.71, *q* = 2.27 × 10^−2^, IVW) increased the risk of renal cell cancer (Table 2 and Figure 3).

### 3.6. Renal Pelvis Cancer

In MR analysis at the *p* < 5 × 10^−8^ level, we found that the genus *Eubacterium coprostanoligenes group* (OR = 146.89, 95% CI = 1.95–11,058.66, *p* = 2.36 × 10^−2^, Wald ratio) increased the risk of renal pelvis cancer (Table 1).

In MR analysis at the *p* < 1 × 10^−5^ level, we found that the family *Clostridiaceae 1* (OR = 0.47, 95% CI = 0.30–0.75, *q* = 3.98 × 10^−3^, IVW) and the genus *Clostridium sensu stricto 1* (OR = 0.55, 95% CI = 0.36–0.84, *q* = 1.73 × 10^−2^, IVW) reduced the risk of renal pelvis cancer; the family *Christensenellaceae* (OR = 2.11, 95% CI = 1.38–3.25, *q* = 2.21 × 10^−3^, IVW) and the genus *Eubacterium eligens group* (OR = 2.43, 95% CI = 1.38–4.26, *q* = 6.33 × 10^−3^, IVW) increased the risk of renal pelvis cancer (Table 2 and Figure 3).

### 3.7. Testicular Cancer

In MR analysis at the *p* < 5 × 10^−8^ level, we found that family *Peptostreptococcaceae* (OR = 14.48, 95% CI = 1.22–171.37, *p* = 3.40 × 10^−2^, Wald ratio) and the genus *Romboutsia* (OR = 14.10, 95% CI = 1.22–162.84, *p* = 3.40 × 10^−2^, Wald ratio) increased the risk of testicular cancer (Table 1).

In MR analysis at the *p* < 1 × 10^−5^ level, we found that the genus *Subdoligranulum* (OR = 0.13, 95% CI = 0.03–0.61, *q* = 1.91 × 10^−2^, IVW) reduced the risk of testicular cancer (Table 2 and Figure 3).

### 3.8. Sensitivity Analyses

Heterogeneity and pleiotropy analyses ensured the reliability and robustness of our MR analysis results. Cochran’s Q statistic of IVW and MR–Egger showed no heterogeneity in five urological cancers (Table 3). The funnel plot suggested the same result as Cochran’s Q statistic (Supplementary Figures S1–S91). The MR–Egger intercept and MR-PRESSO global test showed no potential horizontal pleiotropy (Table 3). We also performed the leave-one-out test and re-analyzed after removing one SNP each time, and our results were still stable (Supplementary Figures S1–S91). Forest plots are shown in Supplementary Figures S1–S91.

### 3.9. Suggestive Associations between Gut Microbiota and Urological Cancers

In MR analysis at the *p* < 1 × 10^−5^ level, we found suggested relationships between some members of the gut microbiota and urological cancers (*p* < 0.05, *q* > 0.05, IVW). The phylum *Bacteroidetes*, order *Desulfovibrionales*, order *NB1n*, family *Family XI*, genus *Lachnospiraceae UCG004*, genus *Victivallis*, genus *Adlercreutzia*, genus *Clostridium sensu stricto 1*, genus *Flavonifractor*, genus *Lachnospiraceae NK4A136 group*, and genus *Romboutsia* were suggested to be related to bladder cancer. The order *Clostridiales*, genus *Eubacterium coprostanoligenes group*, genus *Slackia*, genus *Ruminococcus torques group*, genus *Actinomyces*, genus *Lachnospira*, and genus *Lachnospiraceae UCG008* were suggested to be related to prostate cancer. The *family Clostridiaceae1*, *family Family XI*, *genus Barnesiella*, *genus Slackia*, *genus Alloprevotella*, and *genus Ruminiclostridium 6* were suggested to be related to renal cell cancer. The genus *Coprococcus 2*, genus *Holdemanella*, genus *Howardella*, genus *Ruminiclostridium 5*, genus *Eubacterium brachy group*, genus *Eubacterium eligens group*, genus *Butyricicoccus*, genus *Lactococcus*, and genus *Ruminococcus 1* were suggested to be related to renal pelvis cancer. The family *Ruminococcaceae*, family *Peptostreptococcaceae*, genus *Lachnospiraceae NK4A136 group*, genus *Ruminococcaceae UCG002*, genus *Coprobacter*, and genus *Subdoligranulum* were suggested to be related to testicular cancer.

### 3.10. Reverse MR Analysis

Reverse MR analysis suggested that renal cell cancer may be related to class *Alphaproteobacteria* at *p* < 1 × 10^−5^ level (*p* < 0.05, *q* > 0.05, IVW) (Supplementary Tables S1–S6).

## 4. Discussion

To our knowledge, this study is the first MR analysis to genetically explore the causal relationship between gut microbiota and urologic cancers. Based on the largest GWAS of the gut microbiota, our MR study provides fairly strong genetic evidence that alterations in the abundance of specific gut microbiota play an important role in the occurrence and development of urologic cancers. Using genetic variables as tools, MR analysis largely avoids confounding factors and compensates for the lack of observational studies.

Our study supports previous observational evidence. A metagenomic analysis from China found dysregulated Eubacterium abundance in the gut of bladder cancer patients [39]. In another cohort study, upregulation of Eubacterium abundance in urine was associated with non-muscle invasive bladder cancer [40]. Our findings are identical and link this association specifically to the *Eubacterium coprostanoligenes group* and the *Eubacterium ruminantium group*. ECM1 is a glycoprotein that can induce tumor growth by promoting angiogenesis or enhancing epidermal growth factor signaling [41]. As a regulator of the tumor microenvironment, matrix metalloproteinases can degrade the extracellular matrix and infiltrate tumors into surrounding tissues [42]. ECM1-MMP9 plays an important role in the occurrence and development of tumors [43]. Zhang et al. found that Eubacterium can upregulate ECM1 in bladder tissue, increase the expression of MMP9 through the ERK1/2 phosphorylation pathway, and finally lead to the occurrence and development of bladder cancer [40]. Our study found that *Ruminococcus torques group* and *Ruminococcaceae UCG005* increase the risk of prostate cancer, which is consistent with previous observational studies. Liu et al. found that the abundance of Ruminococcus in the gut of castration-resistant prostate cancer (CRPC) patients was increased [44]. Pernigoni et al. also found that the guts of CRPC patients and mouse models were enriched for Ruminococcus [45]. Ruminococcus belongs to Firmicutes, and its main metabolites are short-chain fatty acids (SCFAs) [46]. SCFAs can promote the production of insulin growth factor 1 (IGF-1) in the whole body and the prostate, and IGF-1 can activate the proliferation of prostate cancer cells through the MAPK and PI3K pathways [47]. Ruminococcus has also been shown to positively correlate with serum testosterone levels, possibly through deglucuronidation of testosterone into a bioactive form and reabsorption [48,49]. Testosterone levels are closely related to the development of prostate cancer [50].

Our MR analysis identified more than 20 gut microbial taxa causally associated with urological cancers, most of which were absent or rarely reported in previous studies. To explore the mechanism of this causal relationship, we interpreted it in several ways. (1) As a compound in the bacterial cell wall, lipopolysaccharide is considered a “danger signal” recognized by the immune system [51]. Gut microbial imbalance can lead to increased LPS, which binds to TLR4 to activate NF-κB, mediates the transcription of stress-related compounds, and increases cancer risk [52]. In addition, higher levels of LPS can cause LPS-endotoxemia to promote carcinogenesis [53]. (2) Gut microbial imbalance can lead to abnormal differentiation of T cells [54]. Regulatory T cells (Tregs) can promote tumor development by silencing the immune clearance of tumor cells [51]. Treg levels may be upregulated during gut ecological dysbiosis, creating a friendly environment for cancer initiation, progression, and metastasis [55]. Gut microbial imbalance induces chronic inflammation in the urinary system through NF-B and mTOR pathways, which can trigger oxidative stress and lead to the accumulation of ROS and NOS [3,56]. (3) Gut microbial imbalance can lead to fluctuations in sex hormone levels, and elevated androgen levels increase the risk of prostate cancer [50]. Elevated estrogen levels may activate polycyclic hydrocarbons, leading to the production of carcinogenic metabolites such as free radical cations that induce DNA damage and increase the risk of cancer [57,58]. Most of the current mechanisms remain at the macroscopic whole gut microbiota level, and more mechanistic studies are needed in the future to explore the role of individual taxa on urologic cancers.

As risk factors for urologic cancers, we propose that obesity and smoking may affect urological cancers by targeting the gut microbiota. Nowadays, a high-fat diet based on carbohydrates and fats is becoming one of the major components of the population’s diet, and the incidence of obesity is increasing significantly [59,60]. The positive relationship between obesity and urologic cancers has been confirmed by several studies, and the mechanisms may be related to insulin resistance, abnormal IGF system, ectopic fat deposition, and microbiome alterations [3]. However, microbiome alterations have only been mentioned in observational studies [61,62,63]. Our study has made a certain contribution to filling the gap in this field. Compared with a healthy population, there are certain alterations in the abundance of specific gut microbes in obesity, which we have shown to be strongly associated with urological cancers. For example, the abundance of *Ruminococcus torques* is increased in the gut of obese people, and *Ruminococcus torques* is associated with an increased risk of prostate cancer and renal cell cancer [61]. *Oscillibacter* is associated with normal body weight and has a protective effect on bladder and prostate cancer [64]. BMI and *Rikenellaceae* show a negative linear relationship, and *Rikenellaceae* may reduce the risk of bladder cancer [65]. The discovery of key gut microbial taxa may open up new avenues for the dietary treatment of cancer. On the other hand, the effect of smoking on urological cancer is surprising. A high-quality MR study demonstrated that smoking leads to a decrease in the abundance of *Ruminococcaceae UCG005*, which we believe increases the risk of prostate cancer [66]. This seems to be contrary to the conventional view that “smoking is a risk factor for cancer”. However, a large prospective study from Sweden also came to the same conclusion that smokers have a lower risk of prostate cancer [67]. In conclusion, the relationship between high-fat diet, smoking, gut microbiota, and urologic cancer is complex, and more prospective studies and mediation MR analyses are needed to explore the associations and mechanisms in the future.

Understanding the bidirectional relationship between gut microbiota and urological cancers may contribute to personalized medicine approaches. Our study provides an emerging approach to large-scale screening for urological cancers. Currently, cystoscopy and bladder tissue biopsy are considered to be the gold standard for bladder cancer diagnosis [68]. However, both examinations are invasive, limiting their use in large-scale screening [7]. In recent years, serum prostate-specific antigen (PSA) has been widely used for screening and early detection of prostate cancer due to its non-invasive advantages [69]. Typically, a prostate biopsy is performed when a patient’s PSA level is greater than 4 ng/mL [70]. However, PSA is organ-specific rather than tumor-specific, leading to increased PSA levels in non-neoplastic lesions of the prostate such as benign prostatic hyperplasia [71]. The low specificity of PSA may lead to overdiagnosis and treatment of some nonneoplastic lesions of the prostate [72]. Diagnosis of renal cell cancer is challenging because renal cell cancer is often clinically silent [73]. The clinical manifestations of early renal cell cancer are variable and nonspecific, and when the renal cancer triad (hematuria, pain, mass) appears, renal cell cancer is usually in an advanced state [74]. In fact, renal cell cancer is usually not diagnosed until distant metastases have developed [75]. As mentioned in the introduction, the value of early screening for urological cancers is tremendous, which will greatly improve the five-year survival rate and prognosis of patients. We believe that it is necessary and feasible to construct a risk assessment model for urological cancers based on changes in the abundance of specific taxa of gut microbiota. The model should take into account the changes in abundance of all taxa that are genetically predicted to be related to urologic cancers, not just a specific taxon. In fact, it has been demonstrated that a risk score based on changes in gut microbiota abundance can diagnose prostate cancer more accurately than PSA examination (area under the curve = 0.81 vs. 0.67) [76].

Our study may pave the way for the development of targeted interventions. When microbes are responsible for the development and progression of urologic cancers, modulating the microbiota may yield potential benefits in the urinary tract [77]. A study including 67 patients with renal cell cancer found that antibiotic use before or after anti-PD-2/PD-L1 immunotherapy resulted in significantly shorter progression-free survival and overall survival [78]. Antibiotic use leads to disturbances in gut microbiota abundance, with changes in Ruminococcus abundance correlating with clinical response to checkpoint inhibition [79]. Another study on differences in gut microbiota composition in prostate cancer patients showed a significant increase in Lachnospiraceae abundance in the gut of patients with oral androgen receptor axis-targeted therapy [80]. Karen et al. suggested that the presence of Lachnospiraceae may be associated with a positive response to anti-PD-1 immunotherapy [80]. Our study suggests that Lactococcus, a significant bacterial model for Lactobacillus, reduces the risk of renal cell cancer. Lactococcus is a potent binder of fibronectin, contributing to the maintenance of barrier homeostasis and moderating inflammatory processes triggered by potentially opportunistic microorganisms and other injuries [81]. The increased strength of fibronectin can induce specific immunity to bladder cancer [82]. We suggest that performing fecal Lactococcus transplantation or direct bladder instillation may induce an immune response to treat bladder cancer. Gut microbiota directly or indirectly involved in the treatment of urological cancers, which may be related to hormones, inflammatory responses, and immunity [77]. Our study identified some gut microbiota associated with reduced risk of urologic cancers, laying the groundwork for translating microbiome research into clinical action and opening avenues for microbial-based therapies. Based on our study, clinicians can target individuals at higher risk of urological cancers based on their gut microbial profile and guide personalized interventions to mitigate risk, such as fecal transplantation, probiotic therapy, and dietary therapy.

Additionally, we provide insights for the optimization of the RCT [83]. (1) RCT is the gold standard for exploring causality [20]. Since gut microbiota plays an irreplaceable role in body homeostasis, it is worthwhile to conduct RCT to explore the role of gut microbiota on urological cancer. Our findings provide a solid theoretical basis for conducting RCTs. (2) Due to the extremely complex composition and a large amount of gut microbiota, it is unrealistic to conduct RCT studies on every taxon. A bidirectional MR study could help identify new avenues of research by revealing a hitherto unknown causal relationship between the gut microbiota and urologic cancers. We screened out a series of specific taxa through gene prediction and determined the cancers that have causal relationships with them. Furthermore, we quantified the risk factors utilizing the OR value and provided references for variables and outcomes within RCTs.

Our study has several limitations. Firstly, the participants in the GWAS for urological cancers were all of the European ancestry, so extrapolation of our results to other ethnic groups requires caution. Secondly, GWAS of the gut microbiota included many ethnicities, which may still result in some bias even though the ancestry of most participants was European (greater than 72.3%). Thirdly, we used the largest GWAS of the gut microbiota to date, but its sample size is still very limited (n = 14,306). In the future, the GWAS of gut microbiota needs to expand the sample size to the traditional GWAS level (n > 100,000) to increase power and reduce errors. Fourthly, urologic cancers are more prevalent in the male population, and the composition of the gut microbiota displays divergence based on gender [2]. Due to the use of summary-level GWAS, our work could not be analyzed separately for the two genders. Gender-specific MR analysis should be carried out in future research. Fifthly, in the process of using drugs to treat diseases, the gut microbiota may develop drug resistance, thus affecting the therapeutic effect. Exploring the drug resistance of taxa that are causally related to urological cancer is meaningful to improve the therapeutic effect of urological cancer. Due to the limitations of the GWAS database, our study cannot explore drug resistance in specific taxa. Therefore, future studies should conduct in-depth studies on drug resistance in the gut microbiota.

## 5. Conclusions

In conclusion, using publicly accessible gene databases, our study comprehensively explored the causal relationship between gut microbiota and five urological cancers. We verified the existing observational evidence and explored the mechanism of gut microbiota on urological cancer from a macroscopic level, providing potential targets for the screening and treatment of urological cancer and new ideas for future clinical research.

## Figures and Tables

**Table 1:** Mendelian randomization (MR) results of significant causal relationship between gut microbiome and urological cancers (*p* < 5 × 10^−8^).

Exposure	Outcome	No. SNP	Methods	β	SE	OR	95% CI	*p*-Value	Causal Direction	Steiger *p*
genus *Allisonella*	Bladder cancer	1	Wald ratio	−0.594	0.202	0.55	0.37–0.82	3.40 × 10^−3^	TRUE	2.60 × 10^−6^
genus *Ruminococcus torques group*	Bladder cancer	1	Wald ratio	1.451	0.513	4.27	1.56–11.68	4.70 × 10^−3^	TRUE	1.54 × 10^−6^
genus *Ruminococcus torques group*	Prostate cancer	1	Wald ratio	0.671	0.258	1.96	1.18–3.25	9.20 × 10^−3^	TRUE	8.62 × 10^−6^
genus *Ruminococcus torques group*	Renal cell cancer	1	Wald ratio	1.414	0.573	4.11	1.34–12.67	1.40 × 10^−2^	TRUE	1.02 × 10^−6^
genus *Eubacterium coprostanoligenes group*	Renal pelvis cancer	1	Wald ratio	4.989	2.204	146.89	1.95–11,058.66	2.40 × 10^−2^	TRUE	4.26 × 10^−7^
family *Peptostreptococcaceae*	Testicular cancer	1	Wald ratio	2.672	1.261	14.48	1.22–171.37	3.40 × 10^−2^	TRUE	2.88 × 10^−6^
genus *Romboutsia*	Testicular cancer	1	Wald ratio	2.646	1.248	14.10	1.22–162.84	3.40 × 10^−2^	TRUE	8.59 × 10^−6^

**Table 2:** Mendelian randomization (MR) results of significant causal relationship between gut microbiome and urological cancers (*p* < 1 × 10^−5^).

Exposure	Outcome	No. SNP	Methods	β	SE	OR	95% CI	*p*-Value	Causal Direction	Steiger *p*
family *Rikenellaceae*	Bladder cancer	18	IVW	−0.372	0.136	0.69	0.53–0.90	6.40 × 10^−3^	TRUE	1.24 × 10^−74^
genus *Eubacterium ruminantium group*	Bladder cancer	18	IVW	0.267	0.090	1.31	1.09–1.56	3.10 × 10^−3^	TRUE	3.21 × 10^−73^
genus *Lachnospiraceae UCG001*	Bladder cancer	11	IVW	−0.302	0.120	0.74	0.58–0.94	1.20 × 10^−2^	TRUE	3.65 × 10^−46^
genus *Oscillibacte*	Bladder cancer	10	IVW	−0.423	0.121	0.66	0.52–0.83	4.80 × 10^−4^	TRUE	2.40 × 10^−40^
genus *Ruminococcaceae UCG013*	Bladder cancer	11	IVW	0.514	0.164	1.67	1.21–2.31	1.80 × 10^−3^	TRUE	3.55 × 10^−46^
genus *Senegalimassilia*	Bladder cancer	4	IVW	0.473	0.162	1.61	1.17–2.21	3.50 × 10^−3^	TRUE	2.83 × 10^−16^
genus *Barnesiella*	Prostate cancer	12	IVW	−1.422	0.485	0.24	0.09–0.62	3.40 × 10^−3^	TRUE	4.18 × 10^−48^
genus *Butyricicoccus*	Prostate cancer	3	IVW	−1.725	0.649	0.18	0.05–0.64	7.90 × 10^−3^	TRUE	1.59 × 10^−20^
genus *Oscillibacter*	Prostate cancer	13	IVW	−0.154	0.057	0.86	0.77–0.96	7.20 × 10^−3^	TRUE	6.46 × 10^−50^
genus *Ruminococcaceae UCG005*	Prostate cancer	14	IVW	0.413	0.150	1.51	1.13–2.03	6.00 × 10^−3^	TRUE	7.84 × 10^−57^
class *Alphaproteobacteria*	Renal cell cancer	6	IVW	0.456	0.192	1.58	1.08–2.30	1.80 × 10^−2^	TRUE	3.89 × 10^−26^
class *Bacilli*	Renal cell cancer	18	IVW	0.342	0.151	1.41	1.05–1.90	2.40 × 10^−2^	TRUE	1.42 × 10^−74^
family *FamilyXI*	Renal cell cancer	8	IVW	−0.273	0.098	0.76	0.63–0.92	5.60 × 10^−3^	TRUE	1.09 × 10^−34^
genus *Coprococcus 2*	Renal cell cancer	9	IVW	−0.468	0.179	0.63	0.44–0.89	9.10 × 10^−3^	TRUE	2.73 × 10^−36^
genus *Eubacterium brachy group*	Renal cell cancer	4	IVW	0.761	0.280	2.14	1.24–3.71	6.60 × 10^−3^	TRUE	6.49 × 10^−18^
genus *Intestinimonas*	Renal cell cancer	16	IVW	−0.374	0.134	0.69	0.53–0.89	5.20 × 10^−3^	TRUE	4.54 × 10^−68^
genus *Lachnoclostridium*	Renal cell cancer	13	IVW	−0.635	0.188	0.53	0.37–0.77	7.70 × 10^−4^	TRUE	5.58 × 10^−52^
genus *Lactococcus*	Renal cell cancer	6	IVW	−0.601	0.244	0.55	0.34–0.88	1.40 × 10^−2^	TRUE	7.62 × 10^−27^
family *Christensenellaceae*	Renal pelvis cancer	9	IVW	0.748	0.218	2.11	1.38–3.25	6.30 × 10^−4^	TRUE	9.96 × 10^−49^
family *Clostridiaceae 1*	Renal pelvis cancer	8	IVW	−0.751	0.234	0.47	0.30–0.75	1.40 × 10^−3^	TRUE	1.89 × 10^−30^
genus *Clostridiumsensustricto 1*	Renal pelvis cancer	7	IVW	−0.601	0.218	0.55	0.36–0.84	6.00 × 10^−3^	TRUE	2.18 × 10^−33^
genus *Eubacterium eligens group*	Renal pelvis cancer	6	IVW	0.886	0.287	2.43	1.38–4.26	2.00 × 10^−3^	TRUE	3.96 × 10^−22^
genus *Subdoligranulum*	Testicular cancer	7	IVW	−2.029	0.781	0.13	0.03–0.61	9.40 × 10^−3^	TRUE	1.42 × 10^−24^

**Table 3:** The results of the sensitivity analyses of MR.

Exposure	Outcome	Method	Heterogeneity		MR-PRESSO	MR–Egger Pleiotropy Test	
			Cochran’s Q	*p*-Value	Global *p*-Value	MR–Egger Intercept	*p*-Value
family *Rikenellaceae*	Bladder cancer	IVW	14.77	0.61	0.62		
		MR–Egger	11.64	0.77		−0.050	0.10
genus *Eubacterium ruminantium group*	Bladder cancer	IVW	17.47	0.42	0.47		
		MR–Egger	17.43	0.36		0.005	0.86
genus *Lachnospiraceae UCG001*	Bladder cancer	IVW	5.31	0.87	0.882		
		MR–Egger	5.30	0.81		−0.005	0.92
genus *Oscillibacte*	Bladder cancer	IVW	9.54	0.39	0.45		
		MR–Egger	9.37	0.31		0.020	0.72
genus *Ruminococcaceae UCG013*	Bladder cancer	IVW	6.44	0.78	0.80		
		MR–Egger	3.48	0.94		−0.062	0.12
genus *Senegalimassilia*	Bladder cancer	IVW	1.79	0.62	0.69		
		MR–Egger	1.43	0.49		0.031	0.61
genus *Barnesiella*	Prostate cancer	IVW	3.70	0.98	0.99		
		MR–Egger	2.64	0.99		−0.138	0.33
genus *Butyricicoccus*	Prostate cancer	IVW	1.17	0.56			
		MR–Egger	1.15	0.28		0.031	0.93
genus *Oscillibacter*	Prostate cancer	IVW	10.60	0.56	0.59		
		MR–Egger	10.59	0.48		−0.002	0.94
genus *Ruminococcaceae UCG005*	Prostate cancer	IVW	16.32	0.23	0.17		
		MR–Egger	12.48	0.41		−0.060	0.08
class *Alphaproteobacteria*	Renal cell cancer	IVW	3.75	0.59	0.56		
		MR–Egger	3.22	0.52		0.046	0.51
class *Bacilli*	Renal cell cancer	IVW	17.61	0.41	0.46		
		MR–Egger	16.40	0.43		−0.033	0.29
family *FamilyXI*	Renal cell cancer	IVW	2.07	0.99	0.96		
		MR–Egger	0.85	0.96		0.091	0.31
genus *Coprococcus 2*	Renal cell cancer	IVW	4.77	0.78	0.80		
		MR–Egger	4.72	0.69		−0.015	0.84
genus *Eubacterium brachy group*	Renal cell cancer	IVW	0.37	0.95	0.95		
		MR–Egger	0.37	0.83		−0.012	0.97
genus *Intestinimonas*	Renal cell cancer	IVW	13.29	0.58	0.61		
		MR–Egger	13.24	0.51		−0.007	0.83
genus *Lachnoclostridium*	Renal cell cancer	IVW	12.97	0.37	0.43		
		MR–Egger	11.87	0.37		−0.044	0.33
genus *Lactococcus*	Renal cell cancer	IVW	4.66	0.46	0.47		
		MR–Egger	3.69	0.45		−0.138	0.38
family *Christensenellaceae*	Renal pelvis cancer	IVW	6.98	0.54	0.58		
		MR–Egger	6.12	0.53		0.036	0.38
family *Clostridiaceae 1*	Renal pelvis cancer	IVW	4.55	0.71	0.74		
		MR–Egger	3.24	0.78		0.065	0.30
genus *Eubacterium eligens group*	Renal pelvis cancer	IVW	3.93	0.56	0.55		
		MR–Egger	2.75	0.60		−0.111	0.34
genus *Clostridiumsensustricto 1*	Renal pelvis cancer	IVW	4.38	0.62	0.65		
		MR–Egger	4.34	0.50		−0.011	0.84
genus *Subdoligranulum*	Testicular cancer	IVW	9.99	0.13	0.18		
		MR–Egger	9.45	0.09		−0.083	0.62

## Data Availability

The summary data of MiBioGen can be downloaded from the website https://mibiogen.gcc.rug.nl/, accessed on 2 April 2023. The summary data of FINNGEN can be downloaded from the website https://www.finngen.fi/en/access_results, accessed on 2 April 2023. The summary data of UK Biobank can be downloaded from the website https://pan.ukbb.broadinstitute.org/, accessed on 2 April 2023. The data presented in this study are available on request from the corresponding author.

## Supplementary Materials

The following supporting information can be downloaded at: https://www.mdpi.com/article/10.3390/nu15184086/s1, Figures S1−S23: The scatter plot of significant gut microbiota on urological cancers; Figures S24−S45: The forest plot of significant gut microbiota on urological cancers; Figures S46−S68: The leave-one-out test of significant gut microbiota on urological cancers; Figures S69−S91: The funnel plot of significant gut microbiota on urological cancers; Table S1: Introduction of Tables S2−S6; Table S2: Selected SNP (*p* < 5 × 10^−8^); Table S3: Selected SNP (*p* < 1 × 10^−5^); Table S4: SNP for significant causal association (*p* < 5 × 10^−8^); Table S5: SNP for significant causal association (*p* < 1 × 10^−5^); Table S6: Potential causal association for reverse MR analysis.

## Abbreviations

CI	confidence interval
RCTs	randomized controlled trials
MR	Mendelian randomization
IVs	instrumental variables
SNPs	single-nucleotide polymorphisms
GWAS	genome-wide association study
LD	linkage disequilibrium
MAF	minor allele frequencies
F	F-statistics
IVW	inverse variance weighted
FDR	false discovery rate
q	q-value
MR-PRESSO	MR Pleiotropy RESidual Sum and Outlier
OR	odds ratio
CRPC	castration-resistant prostate cancer
SCFAs	short-chain fatty acids
IGF-1	insulin growth factor 1
Tregs	regulatory T cells
PSA	prostate-specific antigen
