# Supplementary material for: A Large Genetic Causal Analysis of the Gut Microbiota and Urological Cancers: A Bidirectional Mendelian Randomization Study
Source: Nutrients. 2023 Sep 21;15(18):4086. doi: 10.3390/nu15184086 (PMC10537765; doi:10.3390/nu15184086)
Supplement: Supplementary file 1 [file nutrients-15-04086-s001.zip › nutrients-2597950-supplementary Figure.pdf]

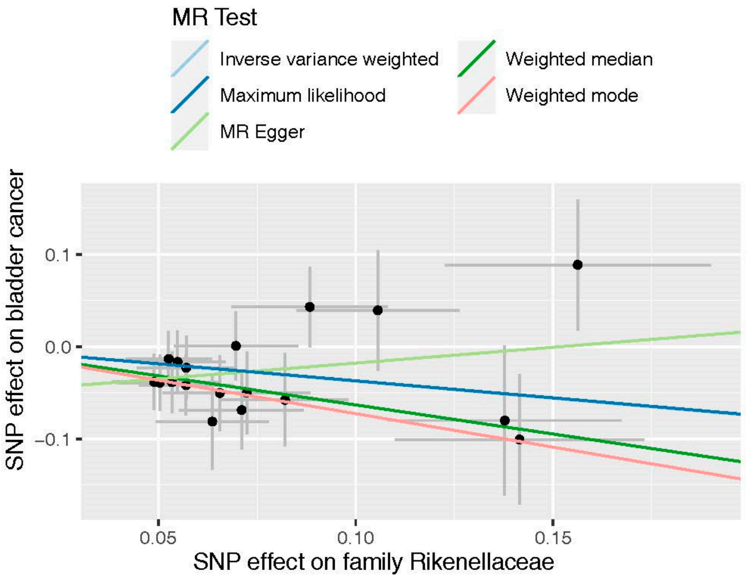

Figure S1. The scatter plot of family Rikenellaceae on bladder cancer

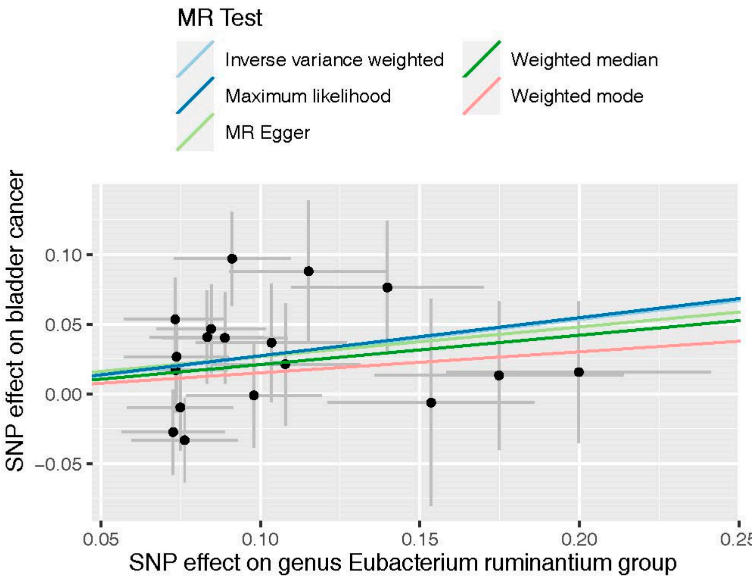

Figure S2. The scatter plot of genus Eubacterium ruminantium group on bladder cancer

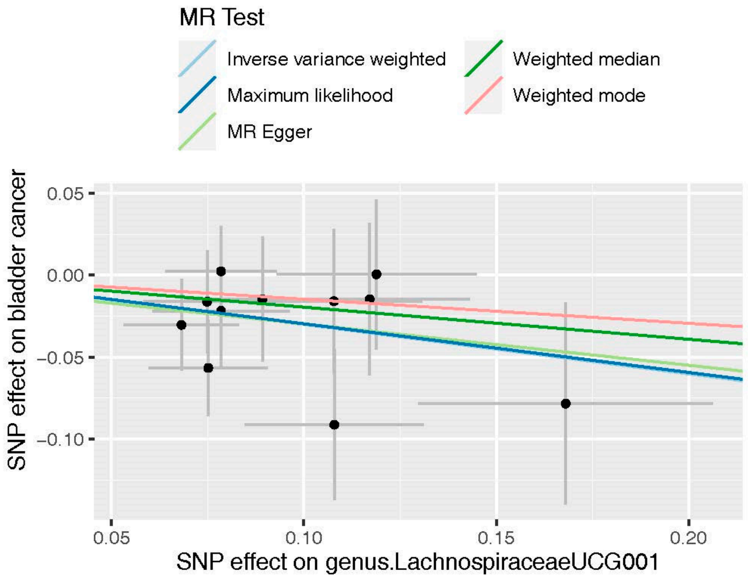

Figure S3. The scatter plot of genus Lachnospiraceae UCG001 on bladder cancer

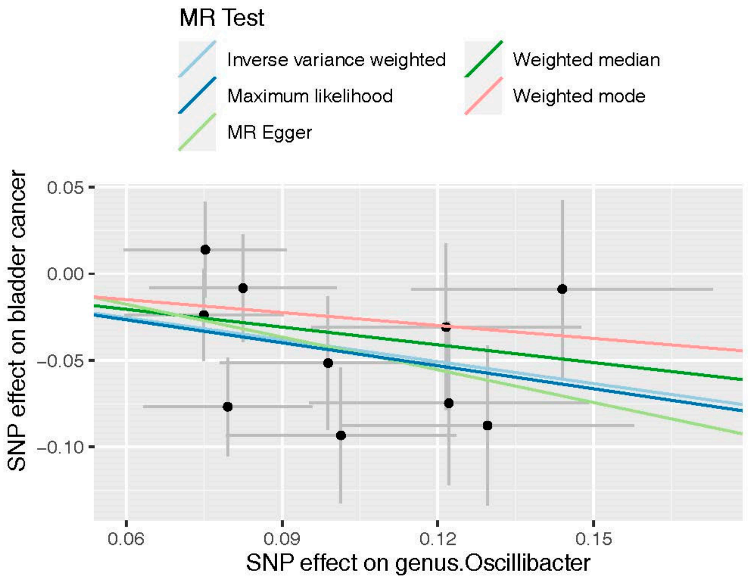

Figure S4. The scatter plot of genus Oscillibacte on bladder cancer

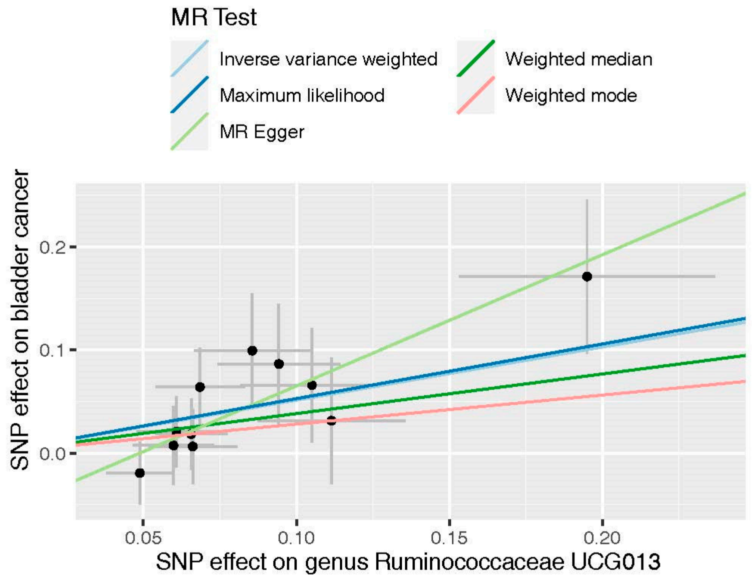

Figure S5. The scatter plot of genus Ruminococcaceae UCG013 on bladder cancer

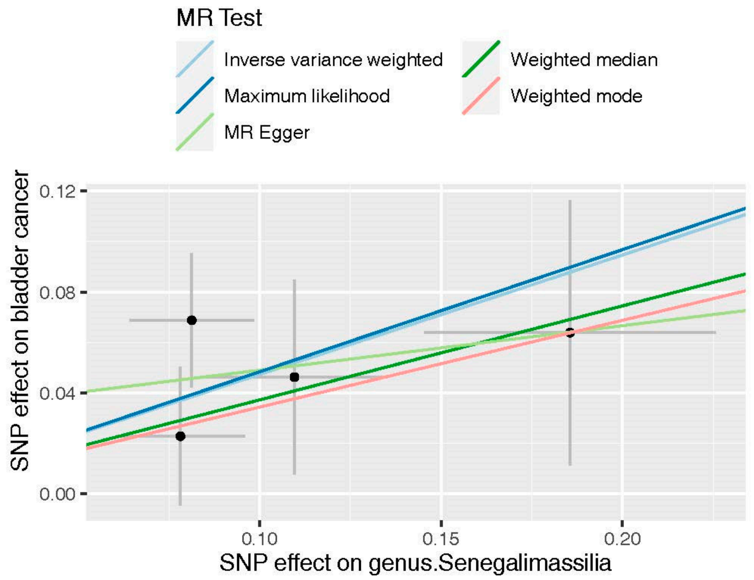

Figure S6. The scatter plot of genus Senegalimassilia on bladder cancer

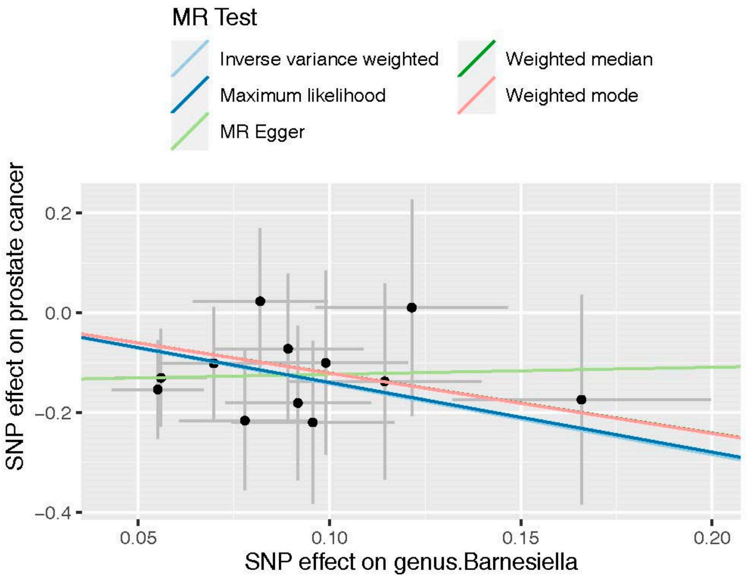

Figure S7. The scatter plot of genus Barnesiella on prostate cancer

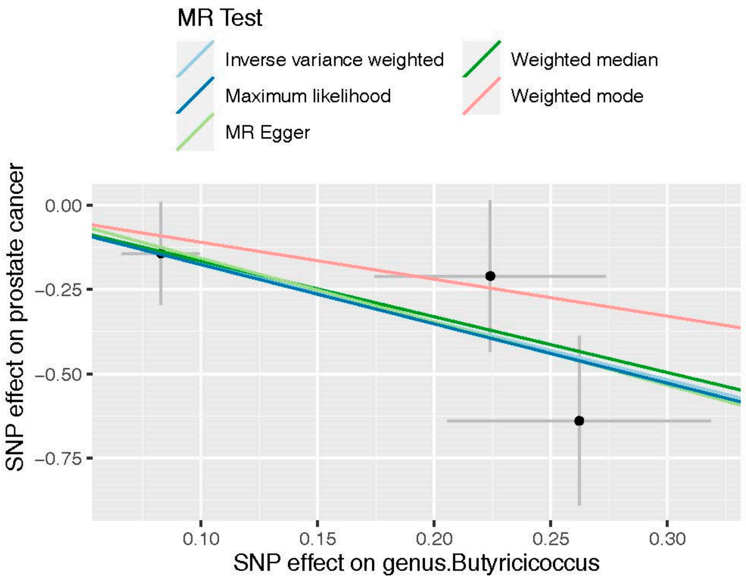

Figure S8. The scatter plot of genus Butyricicoccus on prostate cancer

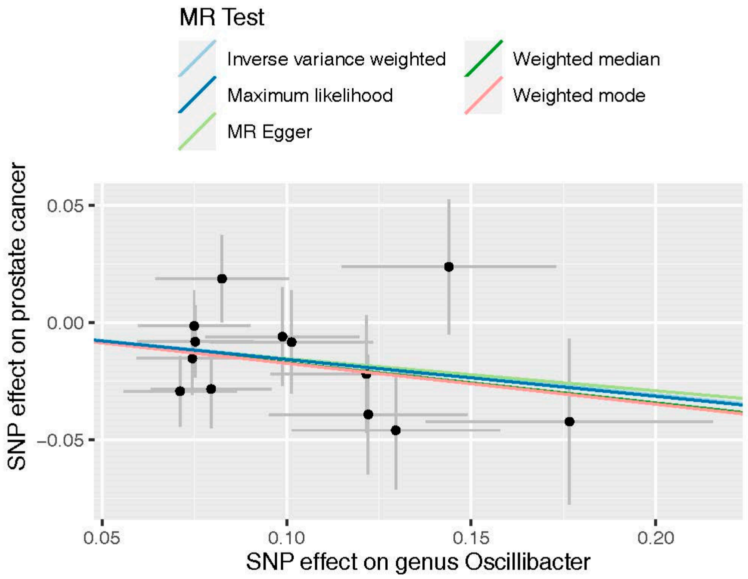

Figure S9. The scatter plot of genus Oscillibacter on prostate cancer

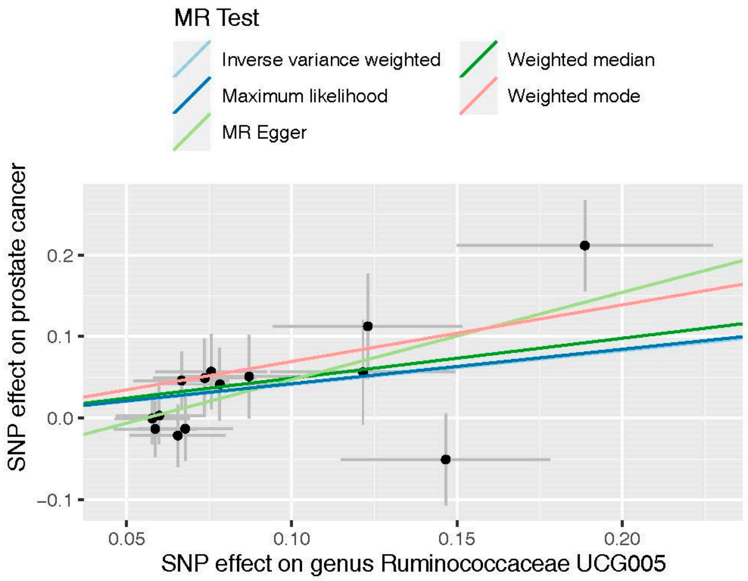

Figure S10. The scatter plot of genus Ruminococcaceae UCG005 on prostate cancer

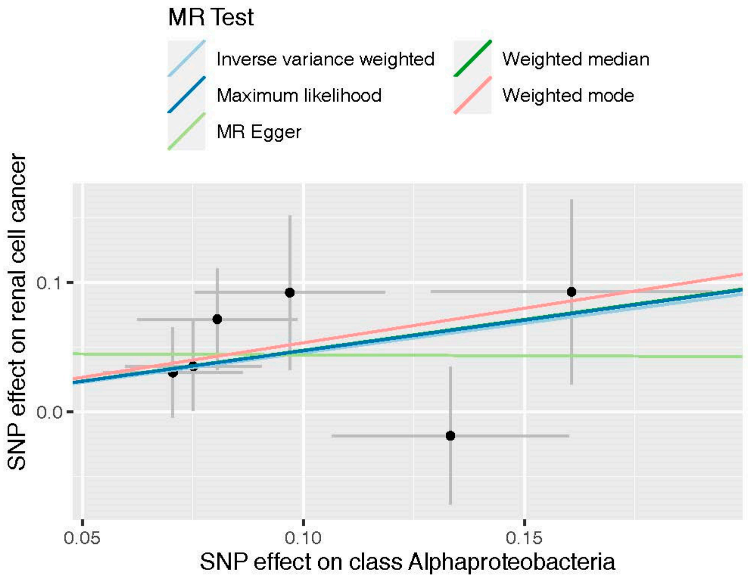

Figure S11. The scatter plot of class Alphaproteobacteria on renal cell cancer

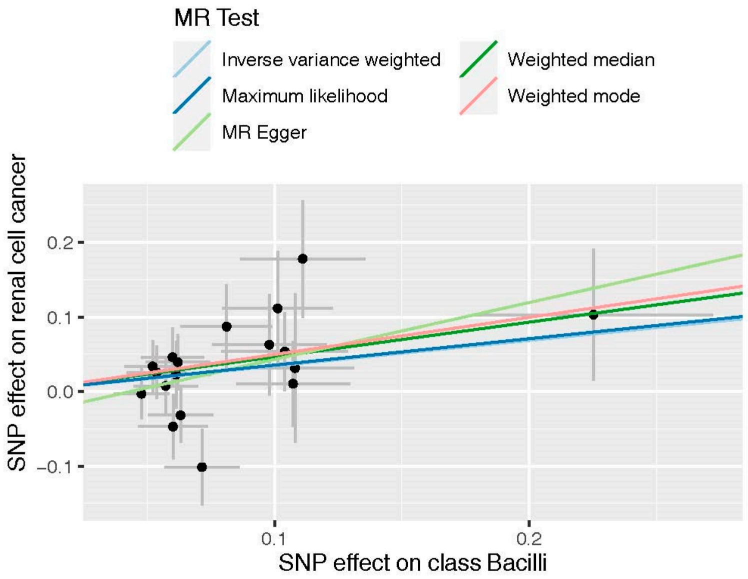

Figure S12. The scatter plot of class Bacilli on renal cell cancer

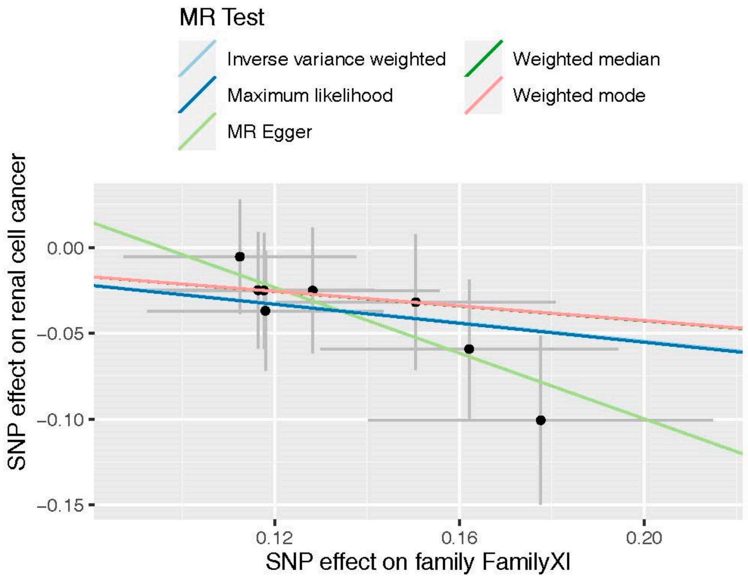

Figure S13. The scatter plot of family FamilyXI on renal cell cancer

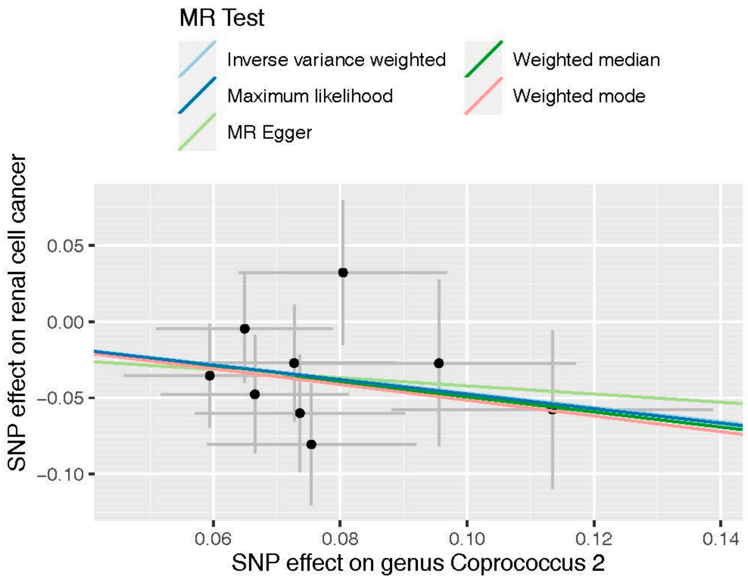

Figure S14. The scatter plot of genus Coprococcus 2 on renal cell cancer

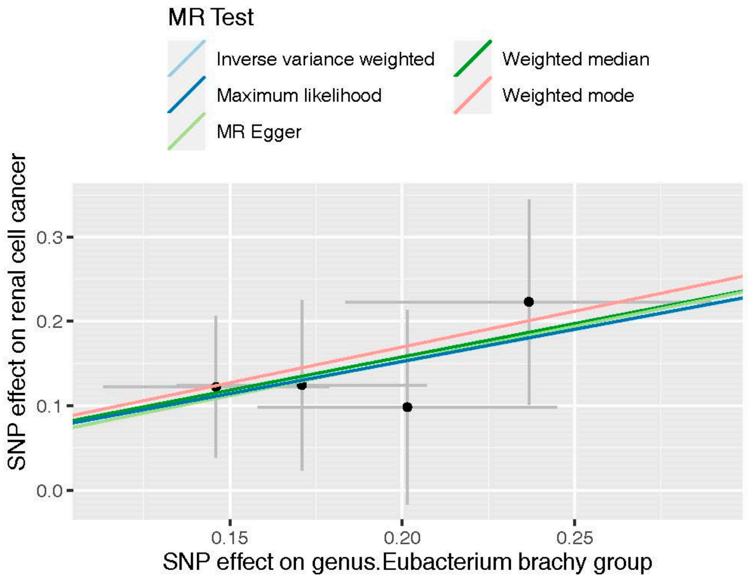

Figure S15. The scatter plot of genus Eubacterium brachy group on renal cell cancer

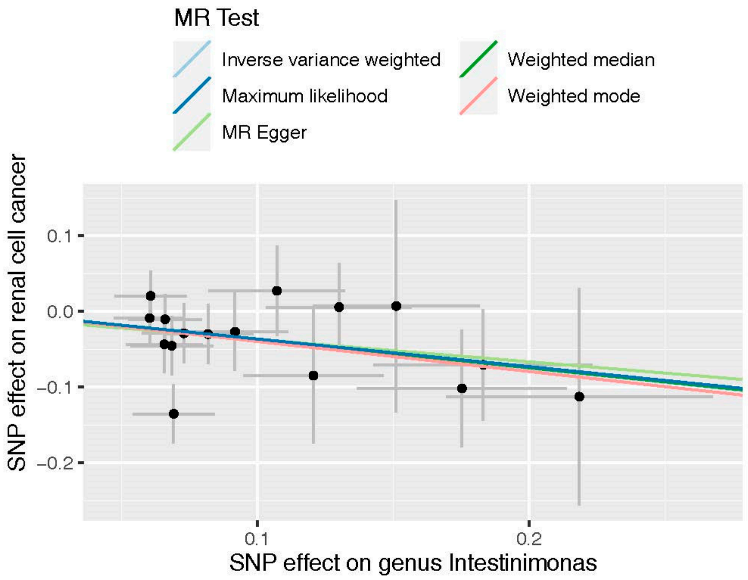

Figure S16. The scatter plot of genus Intestinimonas on renal cell cancer

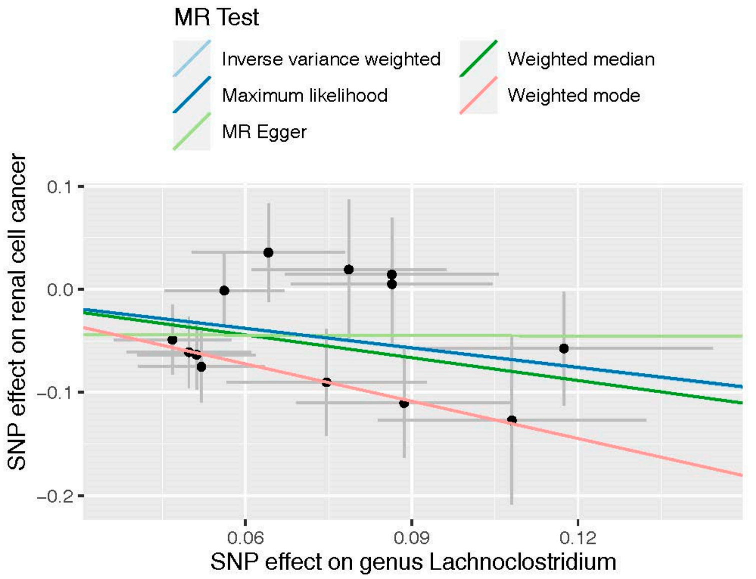

Figure S17. The scatter plot of genus Lachnoclostridium on renal cell cancer

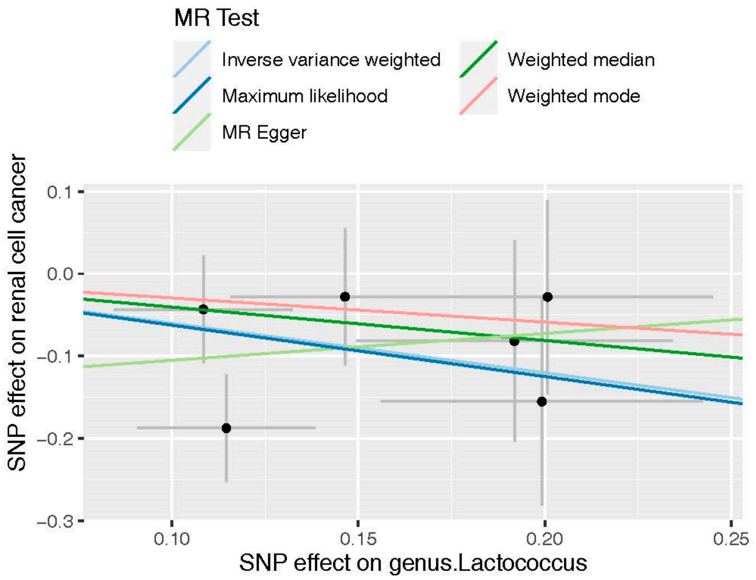

Figure S18. The scatter plot of genus Lactococcus on renal cell cancer

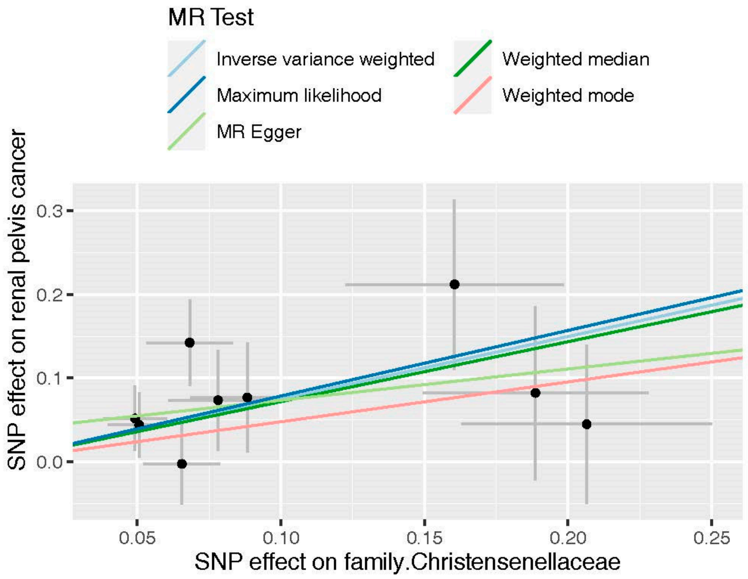

Figure S19. The scatter plot of family Christensenellaceae on renal pelvis cancer

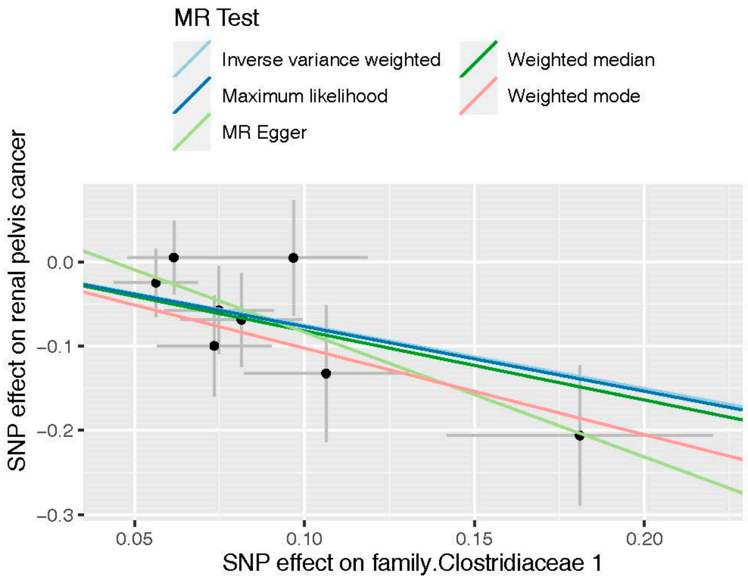

Figure S20. The scatter plot of family Clostridiaceae 1 on renal pelvis cancer

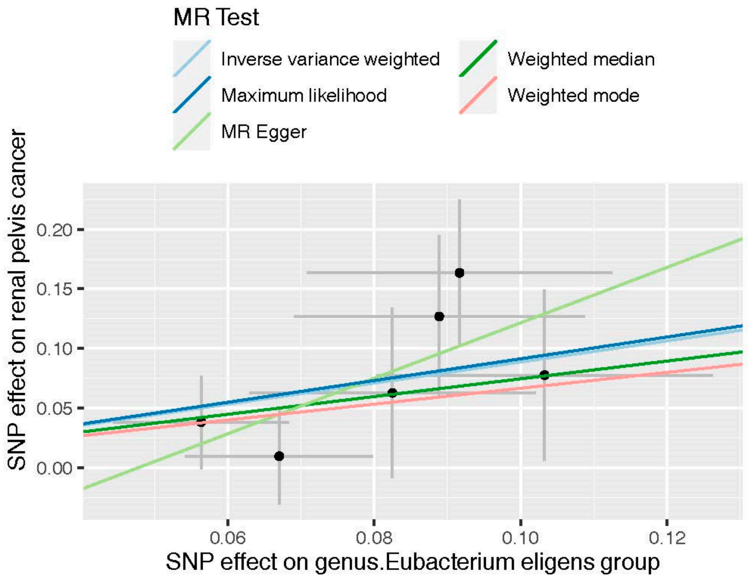

Figure S21. The scatter plot of genus Eubacterium eligens group on renal pelvis cancer

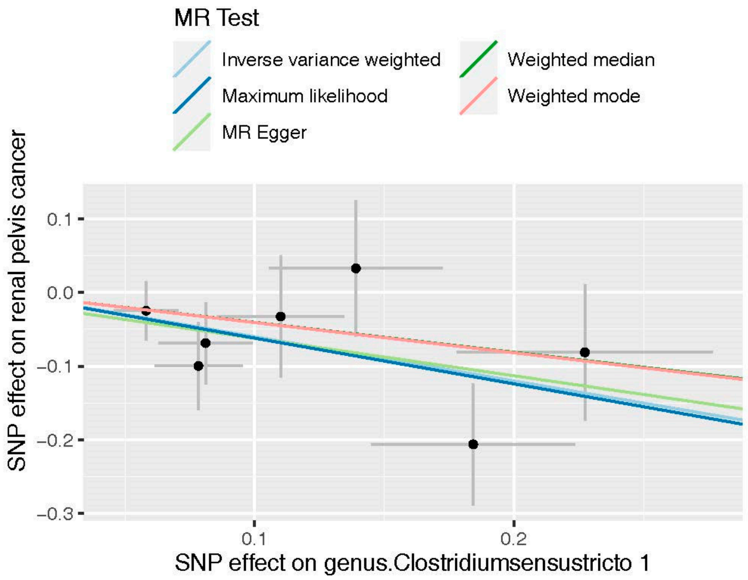

Figure S22. The scatter plot of genus Clostridiumsensustricto 1 on renal pelvis cancer

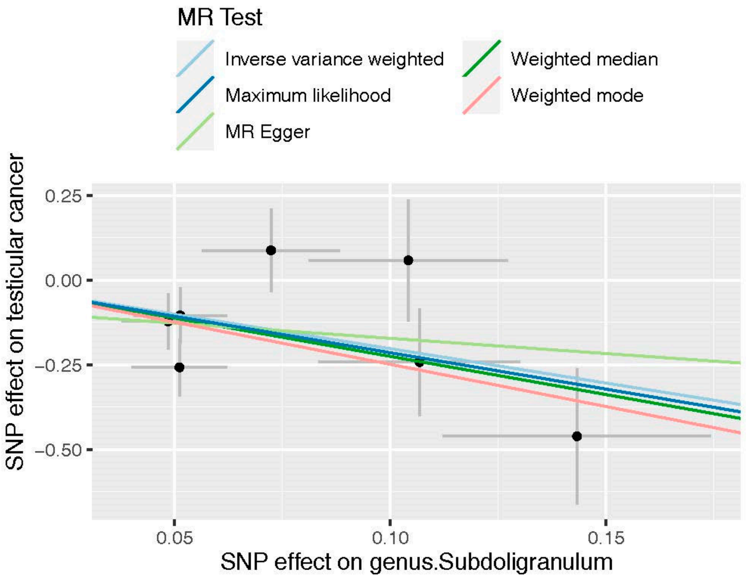

Figure S23. The scatter plot of genus Subdoligranulum on testicular cancer

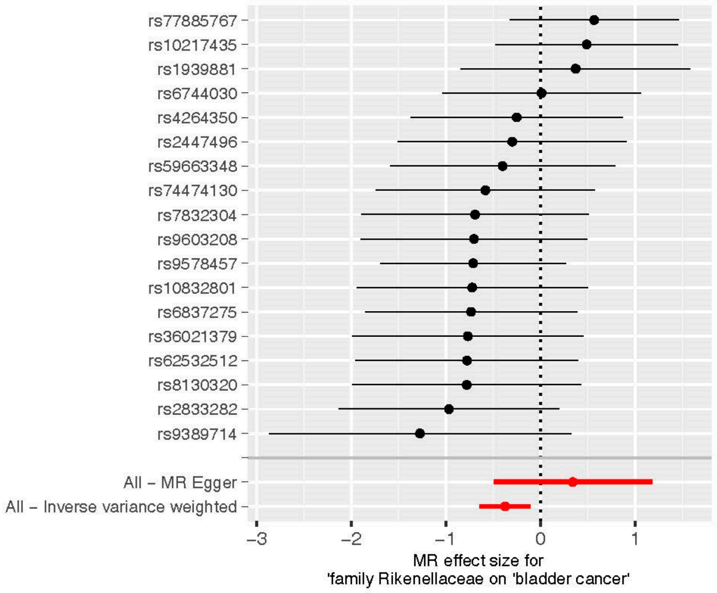

Figure S24. The forest plot of family Rikenellaceae on bladder cancer

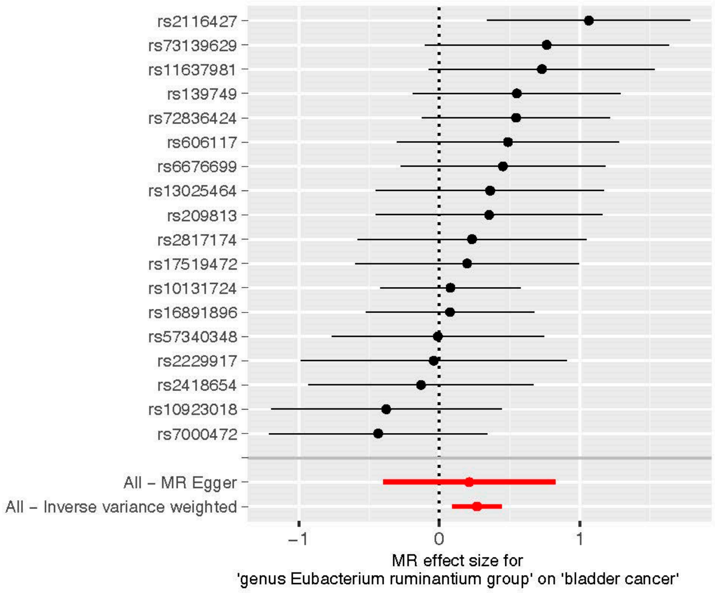

Figure S25. The forest plot of genus Eubacterium ruminantium group on bladder cancer

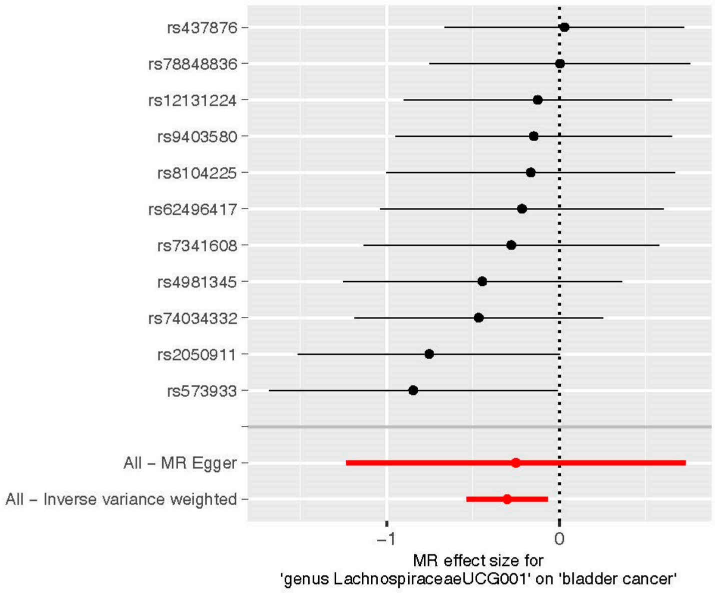

Figure S26. The forest plot of genus Lachnospiraceae UCG001 on bladder cancer

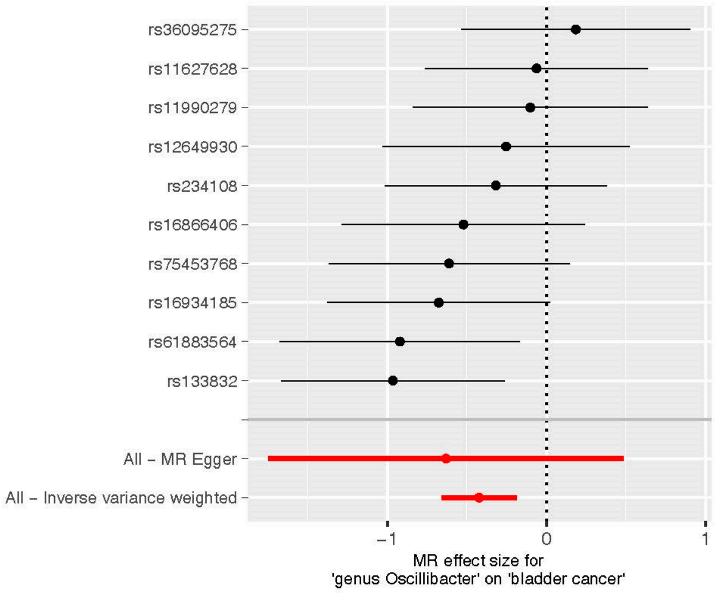

Figure S27. The forest plot of genus Oscillibacte on bladder cancer

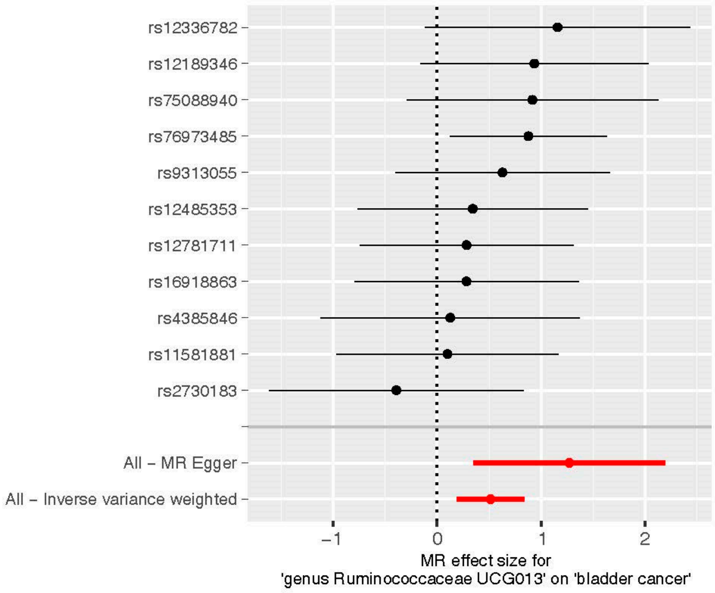

Figure S28. The forest plot of genus Ruminococcaceae UCG013 on bladder cancer

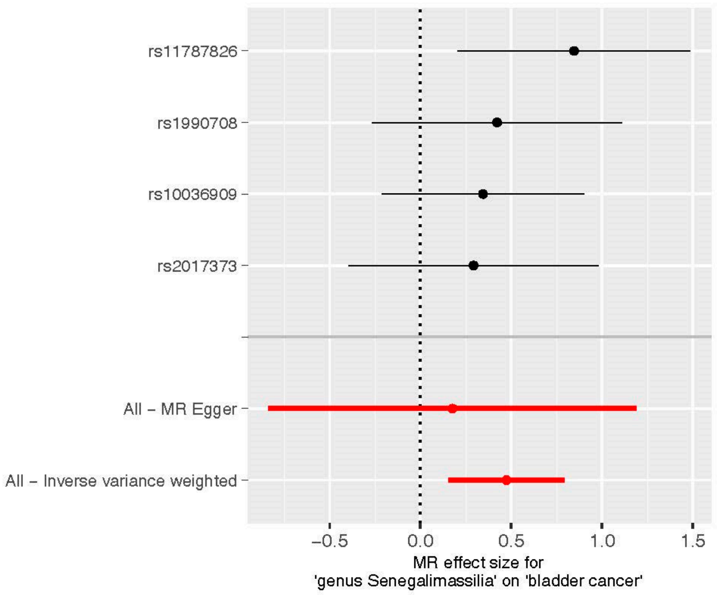

Figure S29. The forest plot of genus Senegalimassilia on bladder cancer

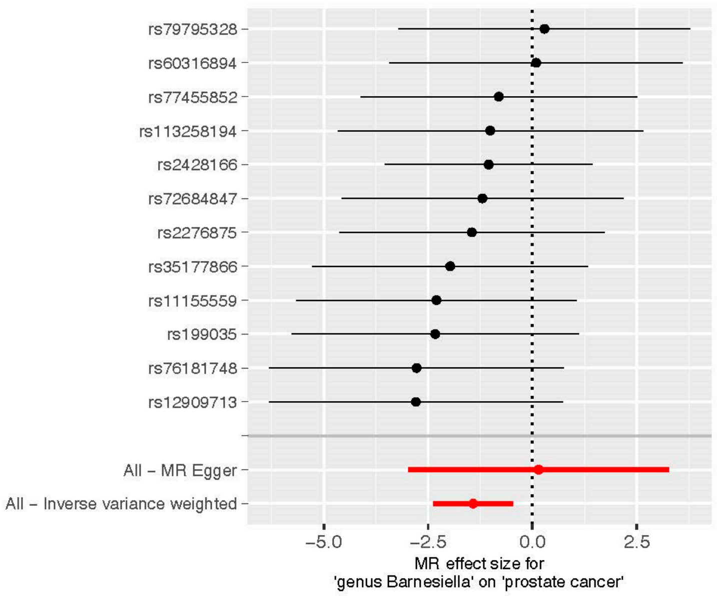

Figure S30. The forest plot of genus Barnesiella on prostate cancer

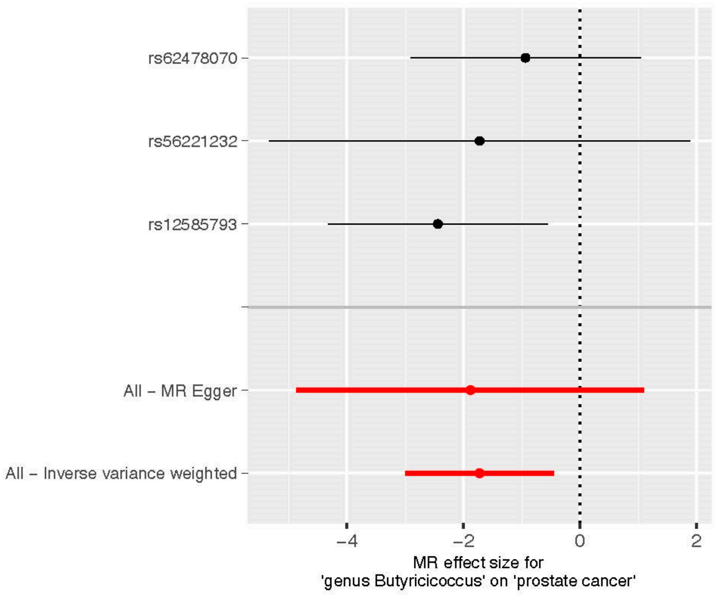

Figure S31. The forest plot of genus Butyricicoccus on prostate cancer

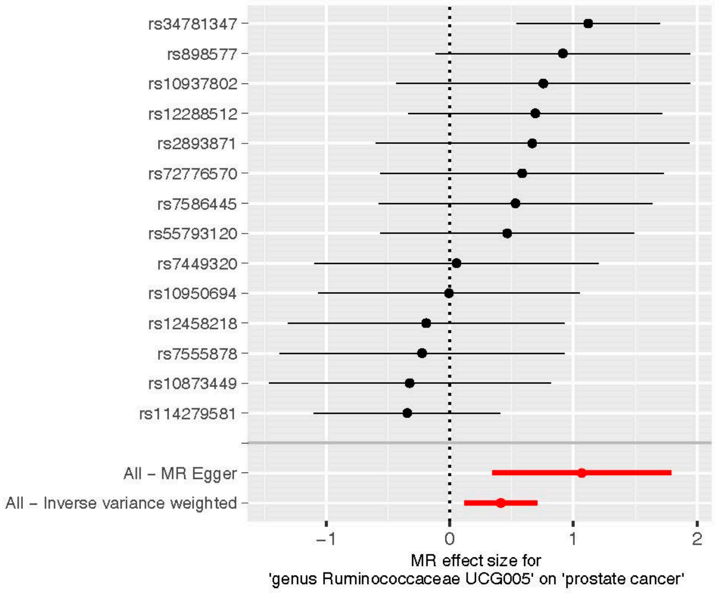

Figure S32. The forest plot of genus Ruminococcaceae UCG005 on prostate cancer

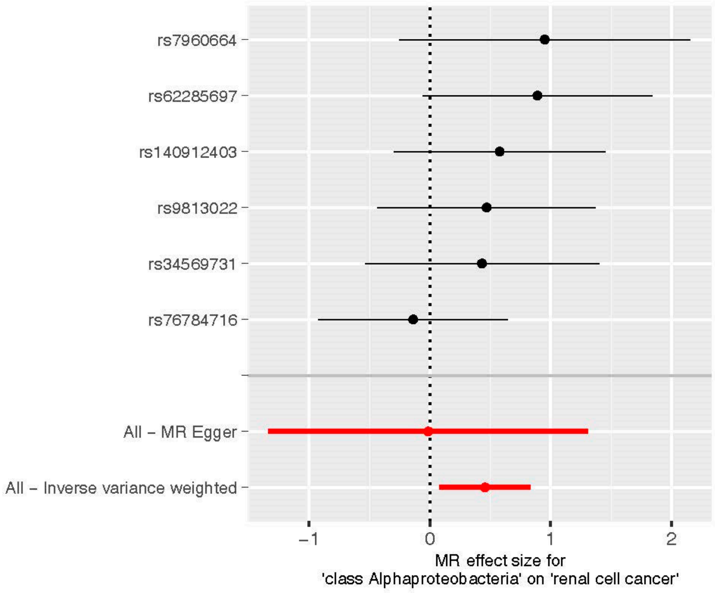

Figure S33. The forest plot of class Alphaproteobacteria on renal cell cancer

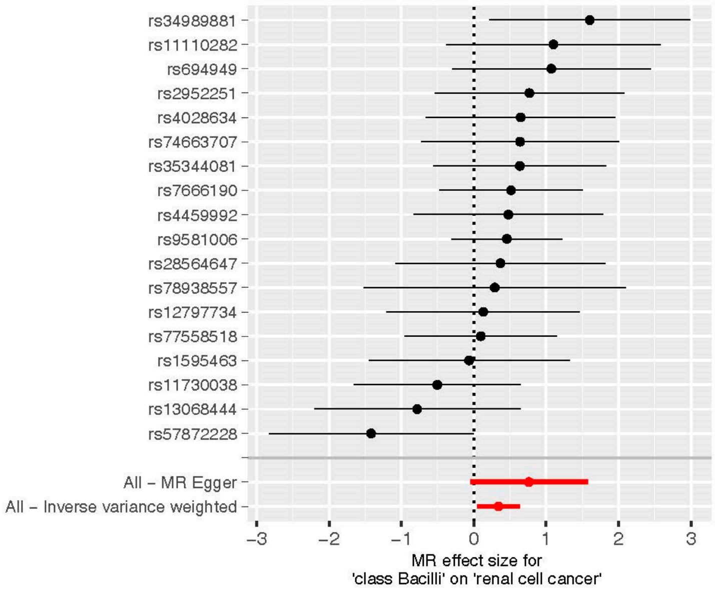

Figure S34. The forest plot of class Bacilli on renal cell cancer

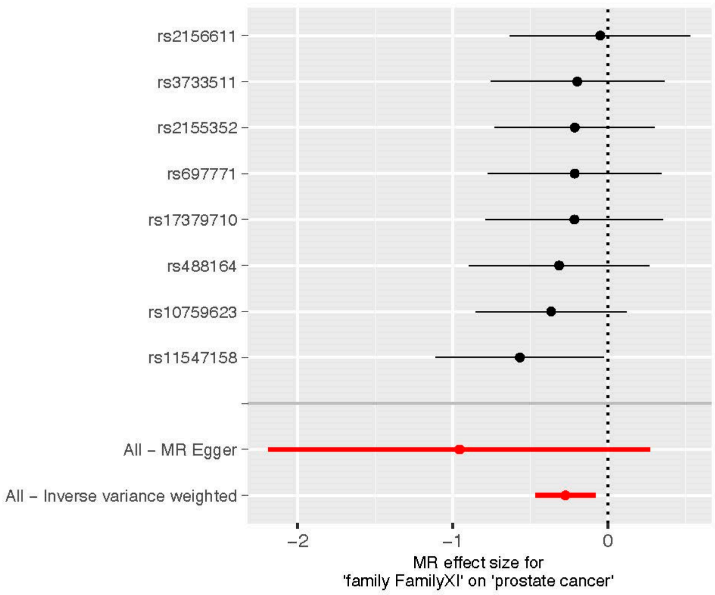

Figure S35. The forest plot of family FamilyXI on renal cell cancer

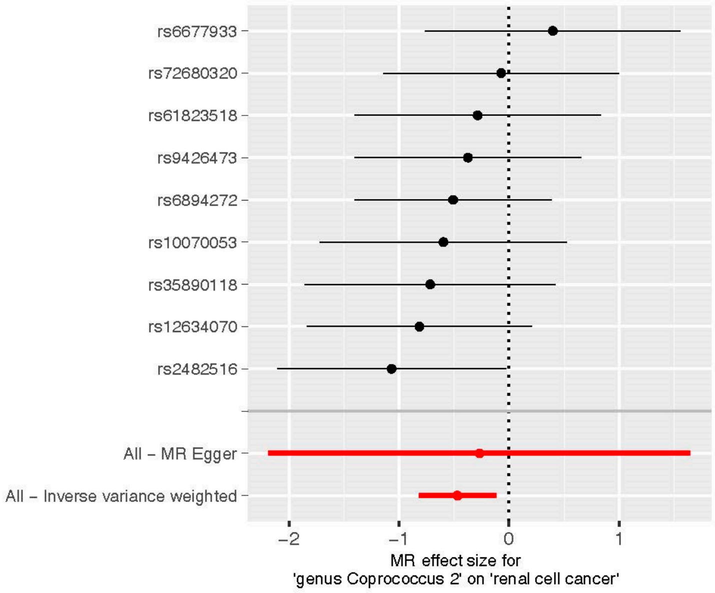

Figure S36. The forest plot of genus Coprococcus 2 on renal cell cancer

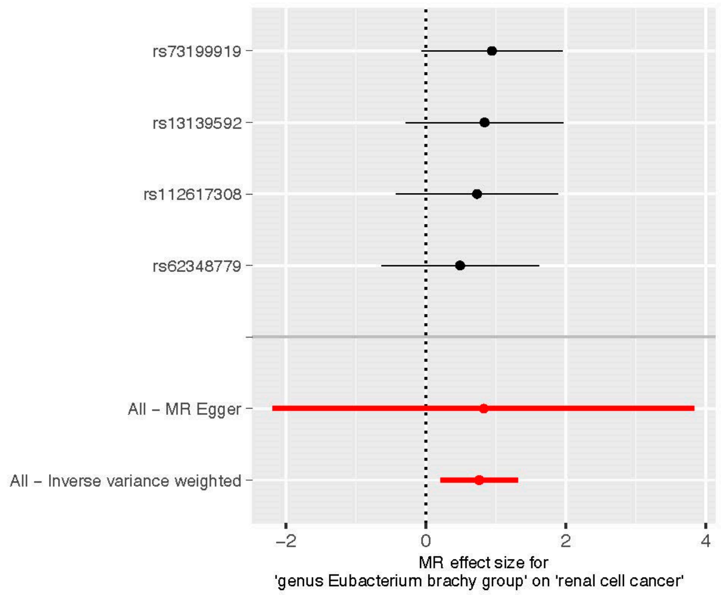

Figure S37. The forest plot of genus Eubacterium brachy group on renal cell cancer

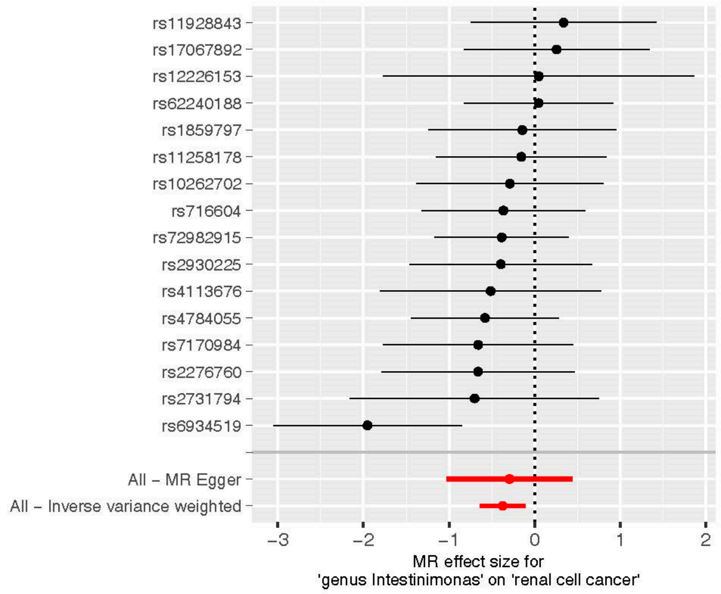

Figure S38. The forest plot of genus Intestinimonas on renal cell cancer

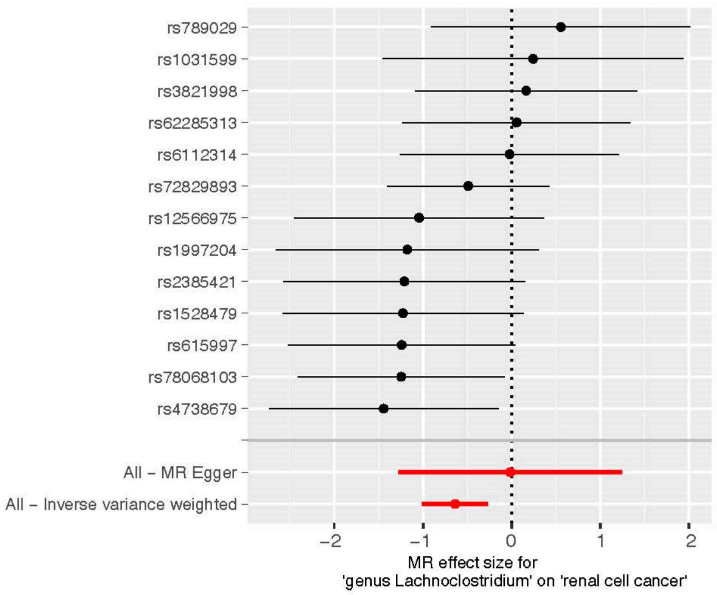

Figure S39. The forest plot of genus Lachnoclostridium on renal cell cancer

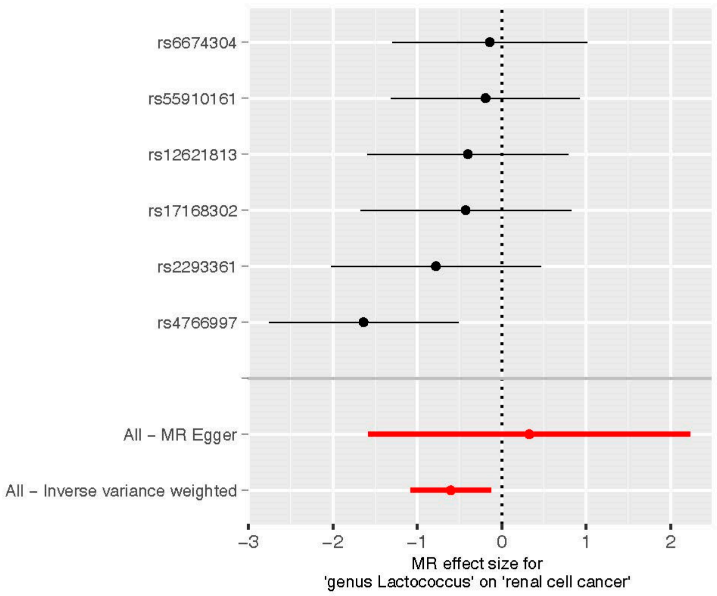

Figure S40. The forest plot of genus Lactococcus on renal cell cancer

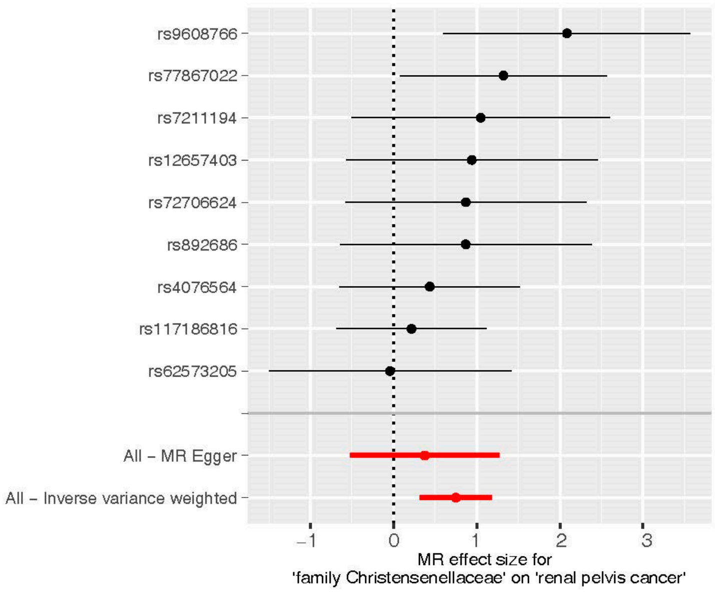

Figure S41. The forest plot of family Christensenellaceae on renal pelvis cancer

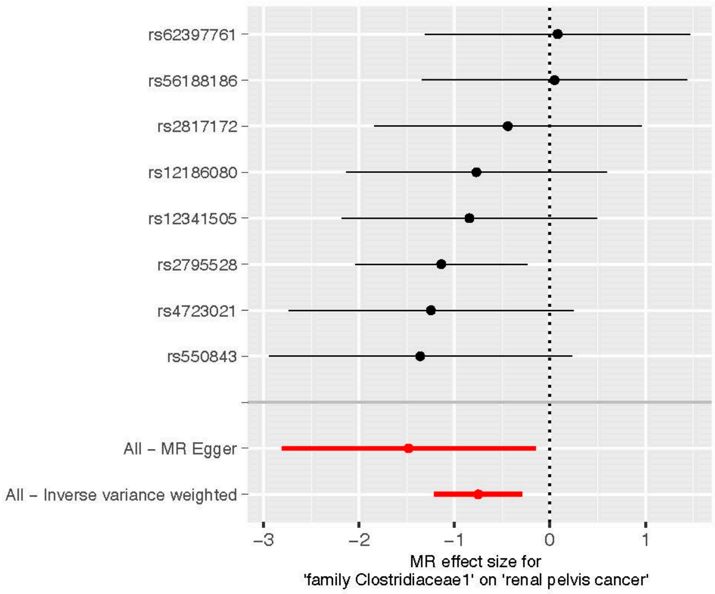

Figure S42. The forest plot of family Clostridiaceae 1 on renal pelvis cancer

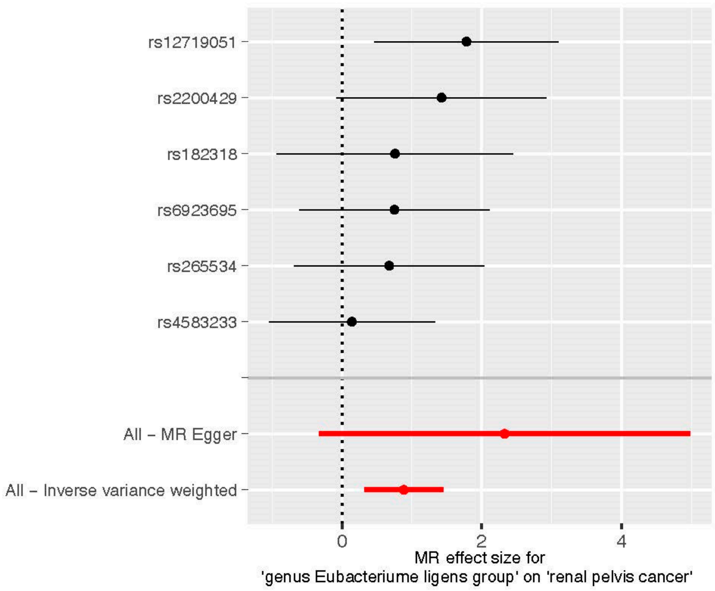

Figure S43. The forest plot of genus Eubacterium eligens group on renal pelvis cancer

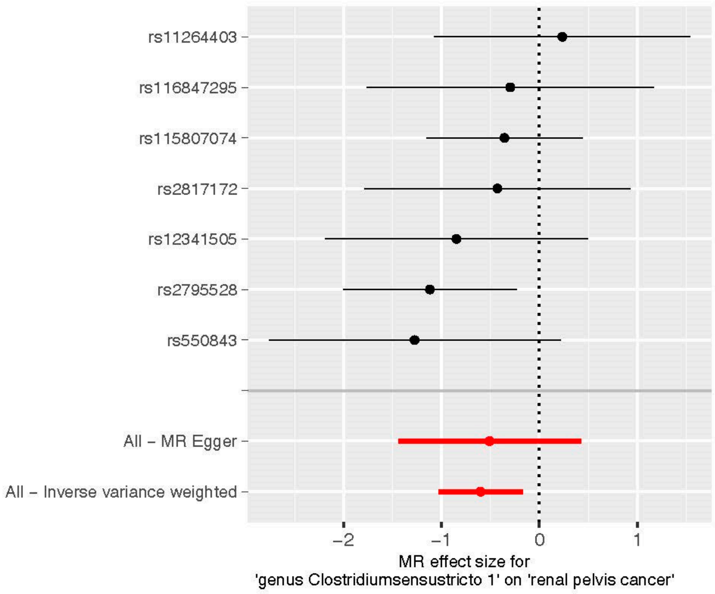

Figure S44. The forest plot of genus Clostridiumsensustricto 1 on renal pelvis cancer

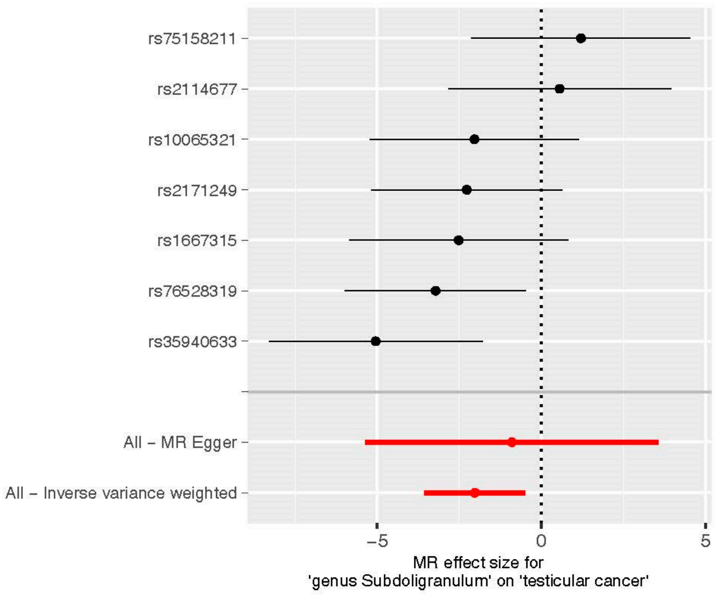

Figure S45. The forest plot of genus Subdoligranulum on testicular cancer

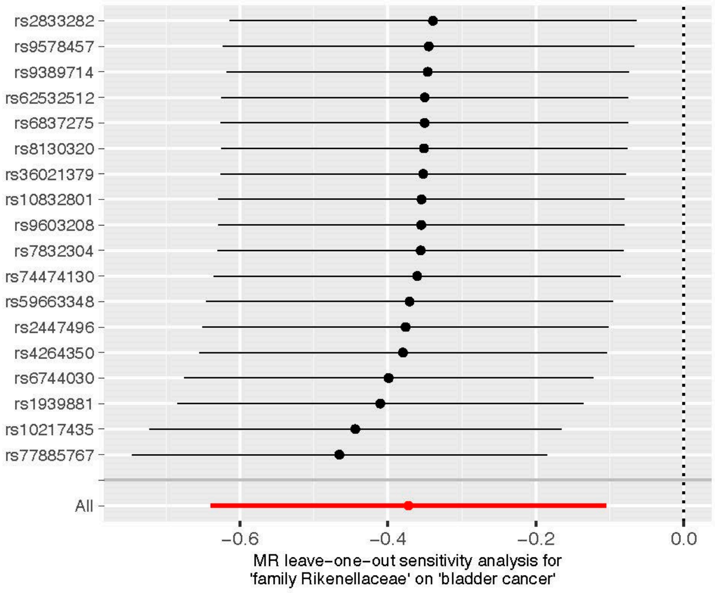

Figure S46. The leave-one-out test of family Rikenellaceae on bladder cancer

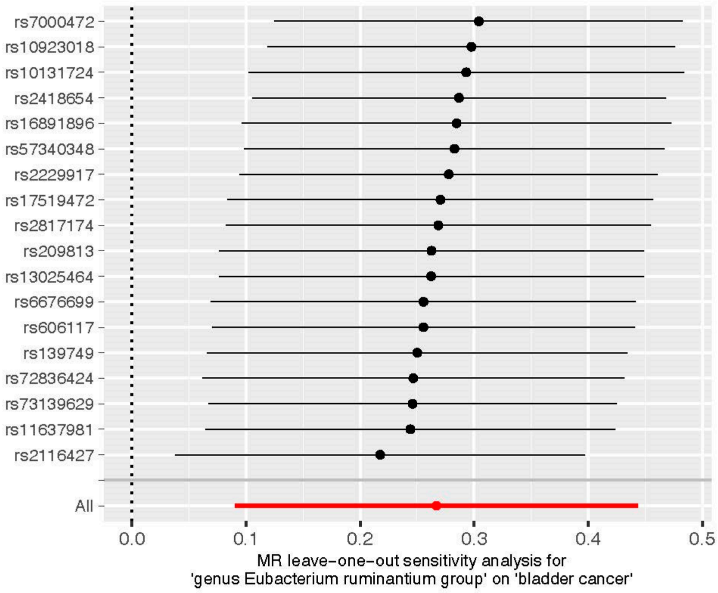

Figure S47. The leave-one-out test of genus Eubacterium ruminantium group on bladder cancer

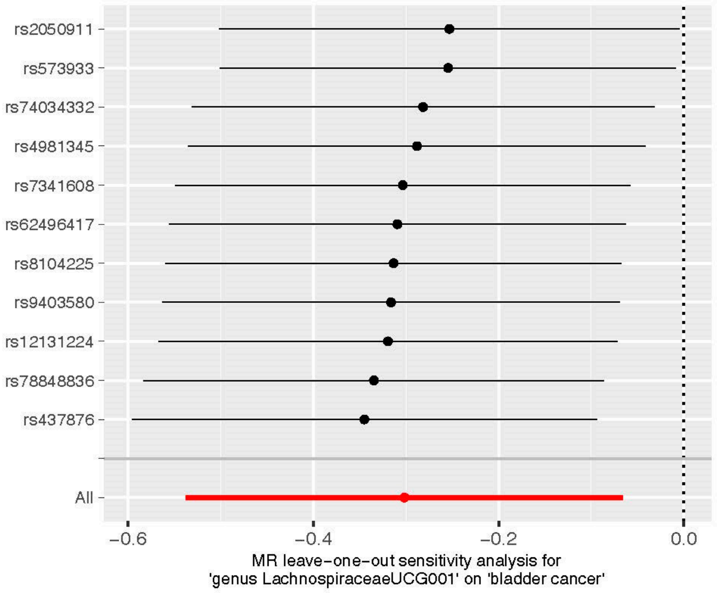

Figure S48. The leave-one-out test of genus Lachnospiraceae UCG001 on bladder cancer

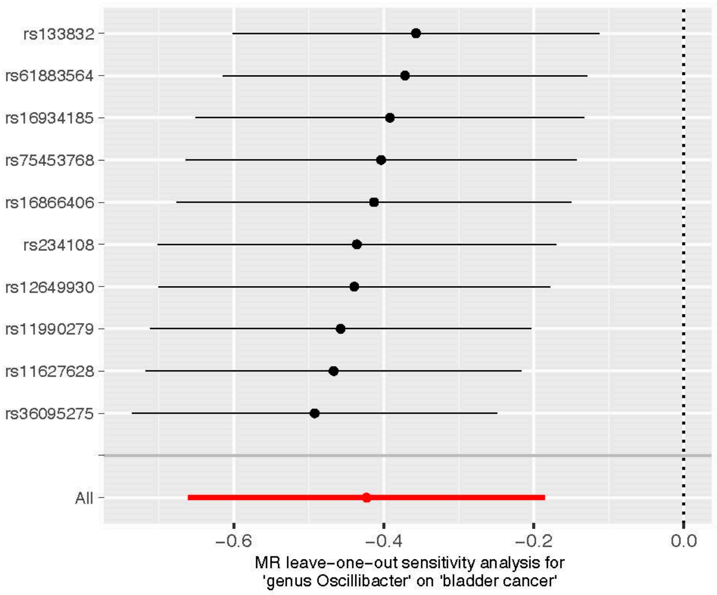

Figure S49. The leave-one-out test of genus Oscillibacte on bladder cancer

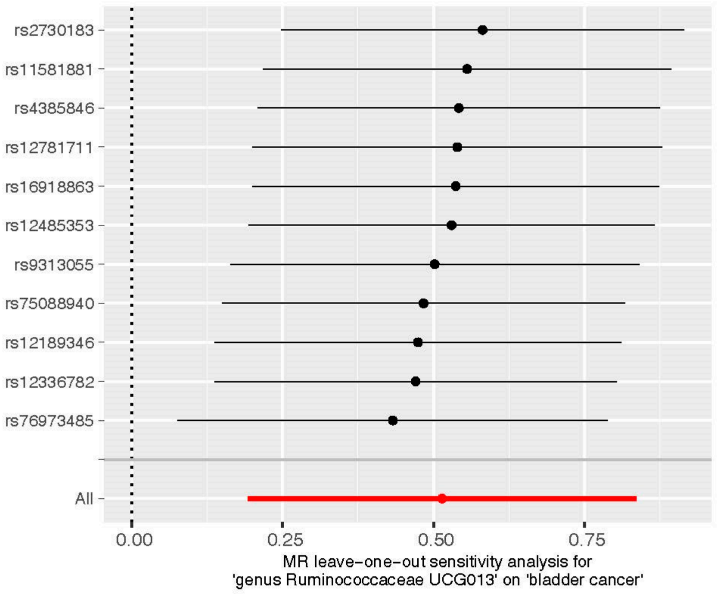

Figure S50. The leave-one-out test of genus Ruminococcaceae UCG013 on bladder cancer

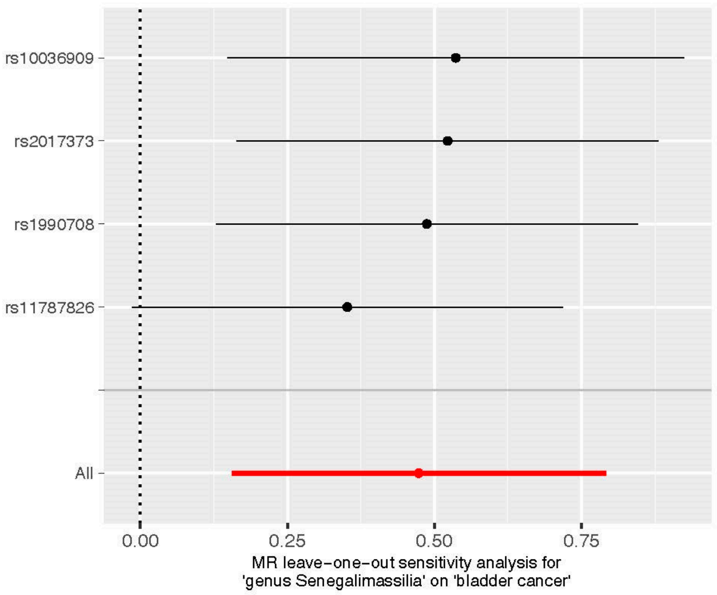

Figure S51. The leave-one-out test of genus Senegalimassilia on bladder cancer

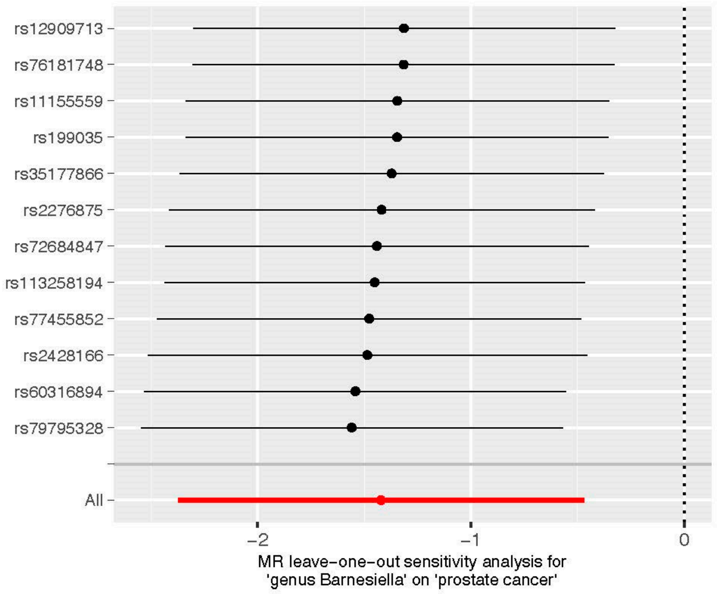

Figure S52. The leave-one-out test of genus Barnesiella on prostate cancer

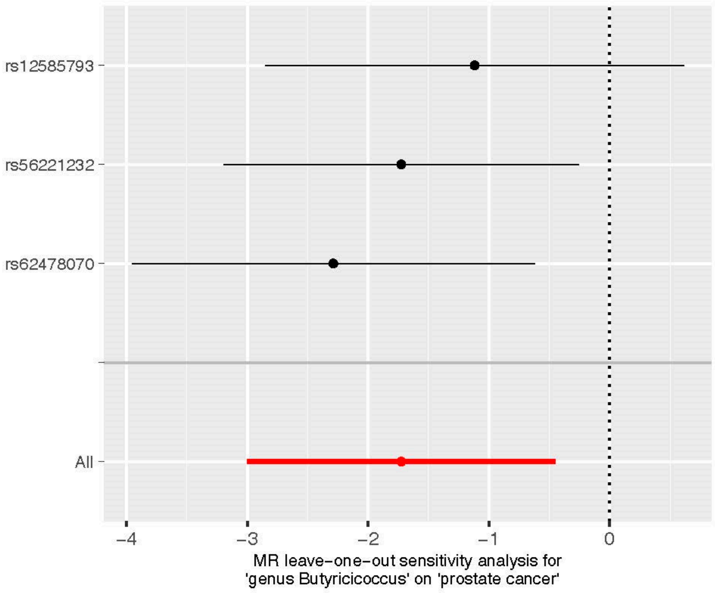

Figure S53. The leave-one-out test of genus Butyricicoccus on prostate cancer

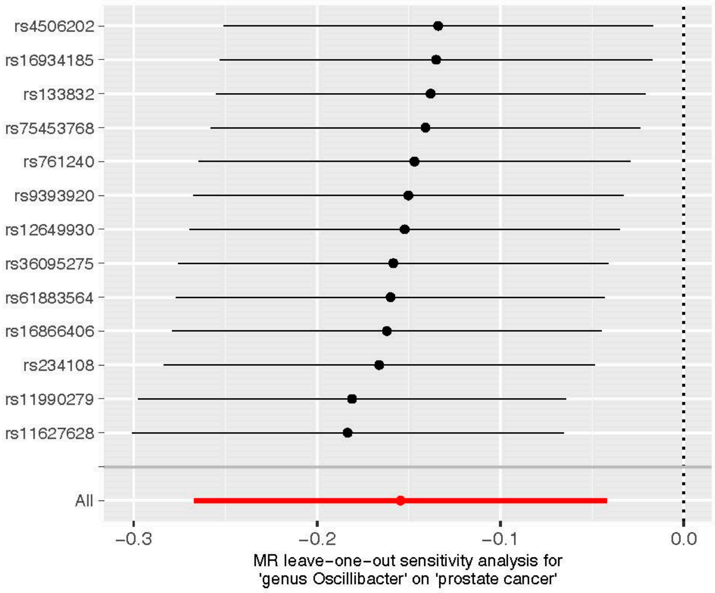

Figure S54. The leave-one-out test of genus Oscillibacter on prostate cancer

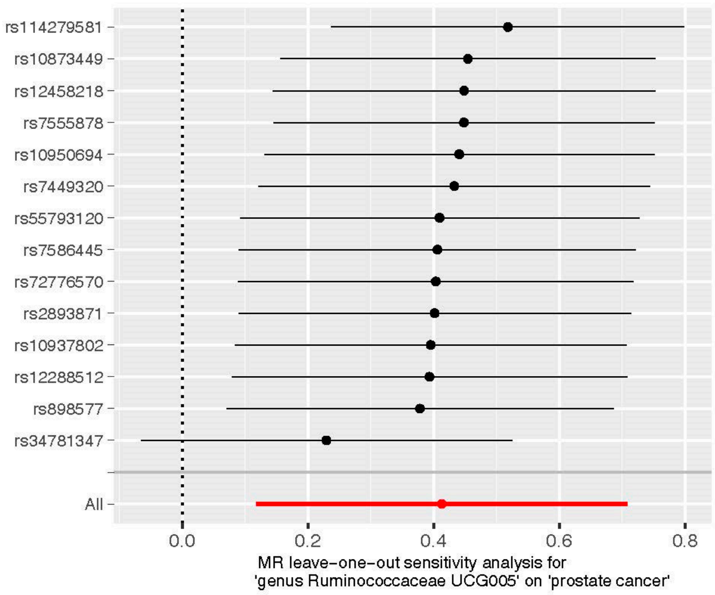

Figure S55. The leave-one-out test of genus Ruminococcaceae UCG005 on prostate cancer

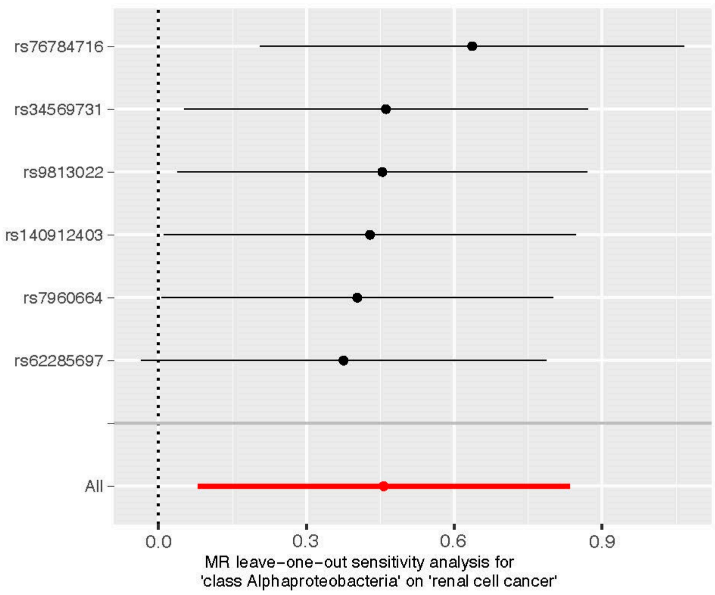

Figure S56. The leave-one-out test of class Alphaproteobacteria on renal cell cancer

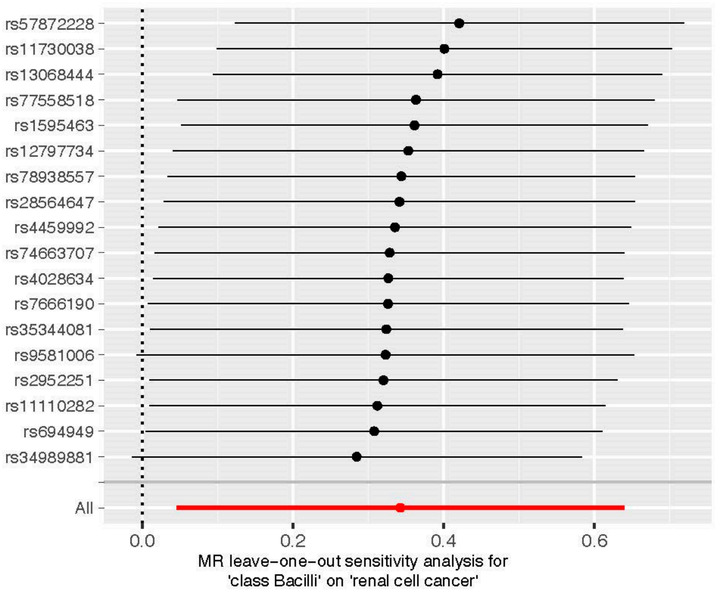

Figure S57. The leave-one-out test of class Bacilli on renal cell cancer

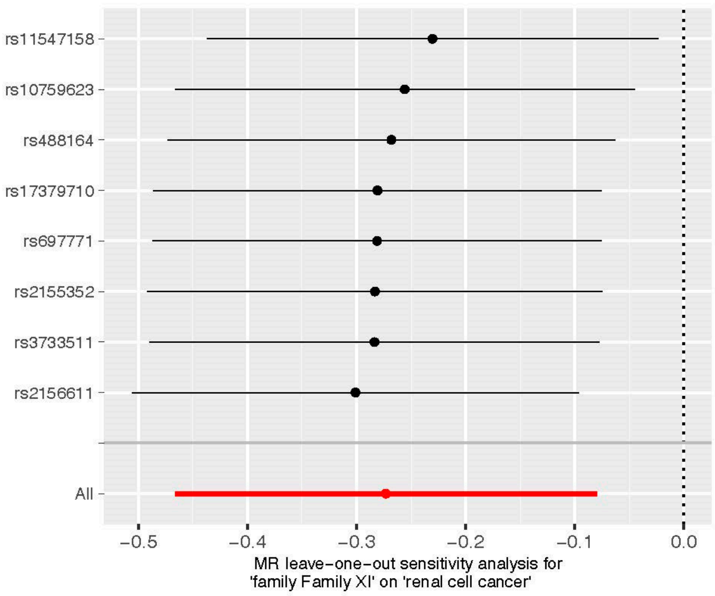

Figure S58. The leave-one-out test of family FamilyXI on renal cell cancer

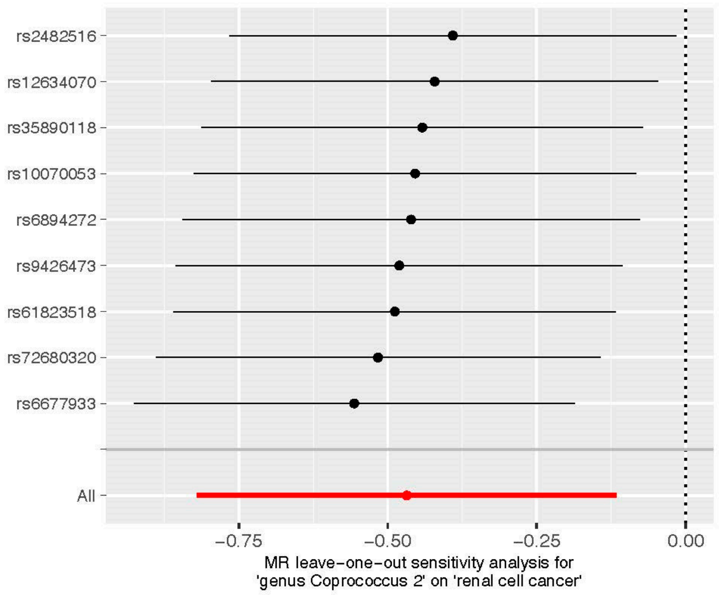

Figure S59. The leave-one-out test of genus Coprococcus 2 on renal cell cancer

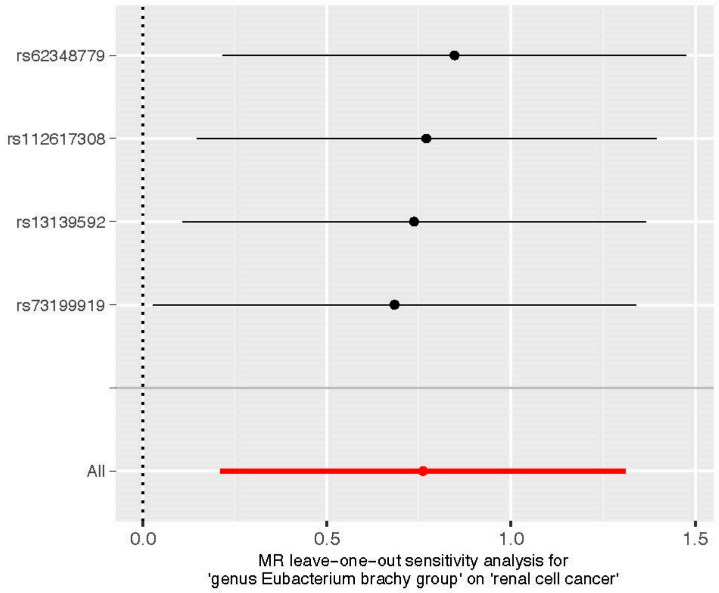

Figure S60. The leave-one-out test of genus Eubacterium brachy group on renal cell cancer

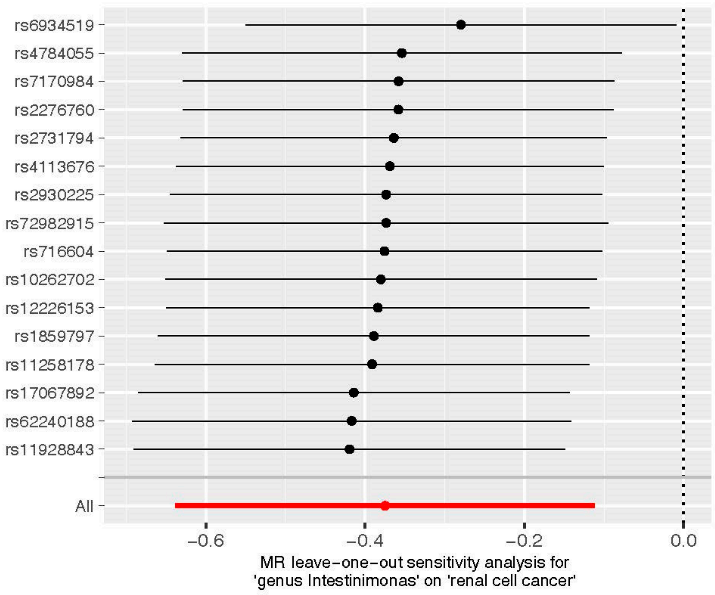

Figure S61. The leave-one-out test of genus Intestinimonas on renal cell cancer

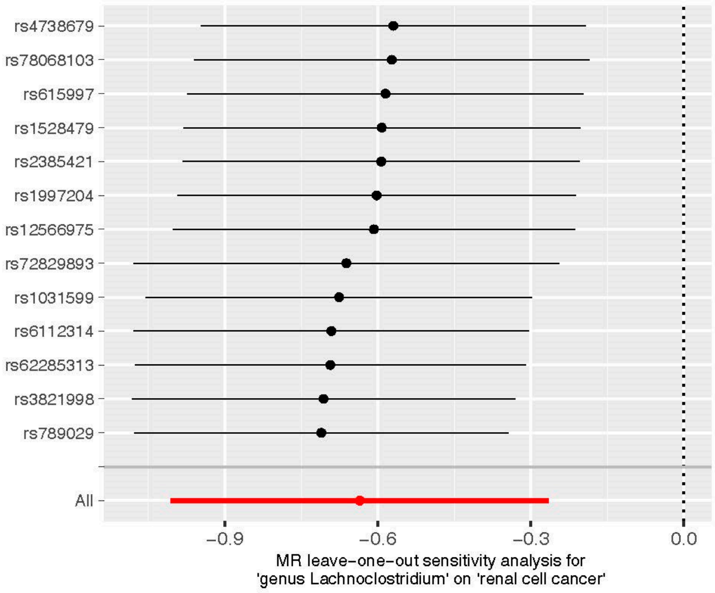

Figure S62. The leave-one-out test of genus Lachnoclostridium on renal cell cancer

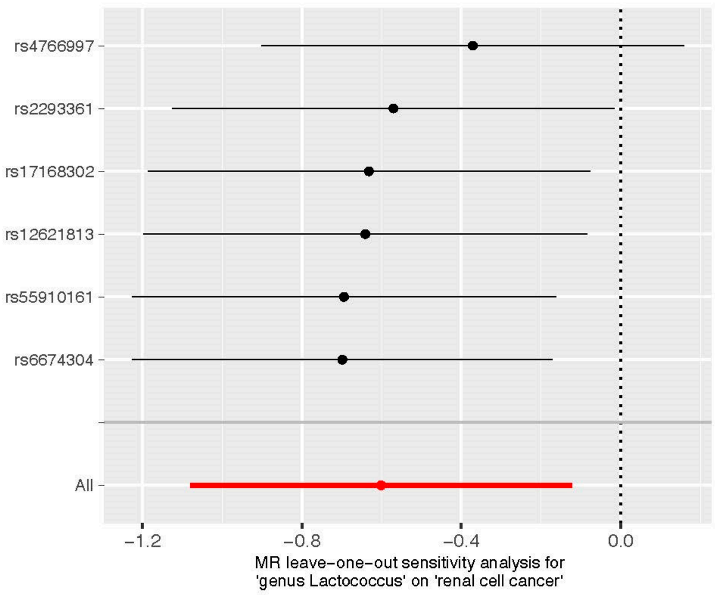

Figure S63. The leave-one-out test of genus Lactococcus on renal cell cancer

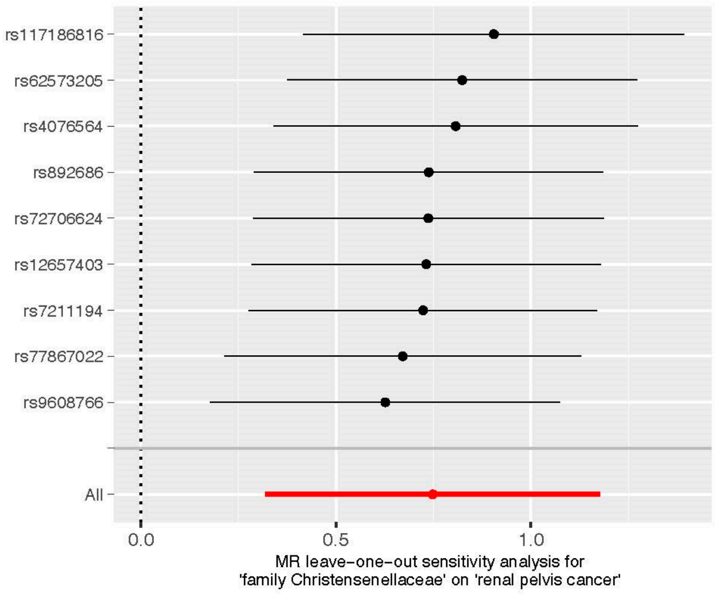

Figure S64. The leave-one-out test of family Christensenellaceae on renal pelvis cancer

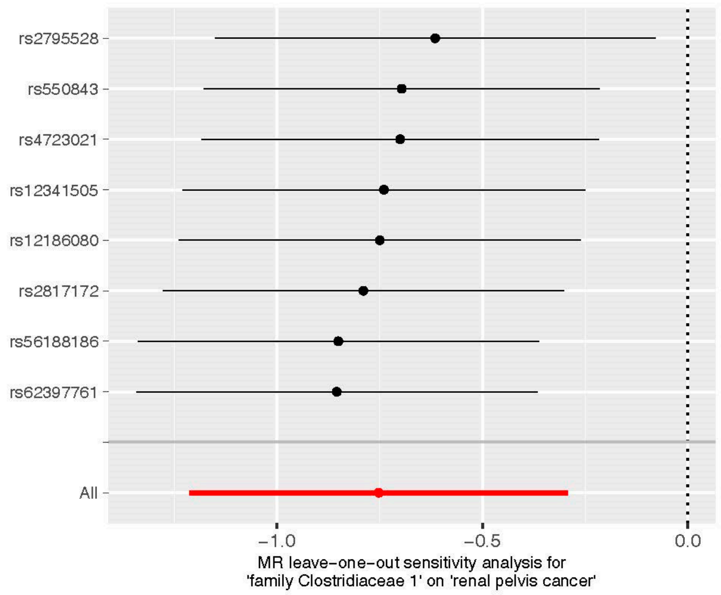

Figure S65. The leave-one-out test of family Clostridiaceae 1 on renal pelvis cancer

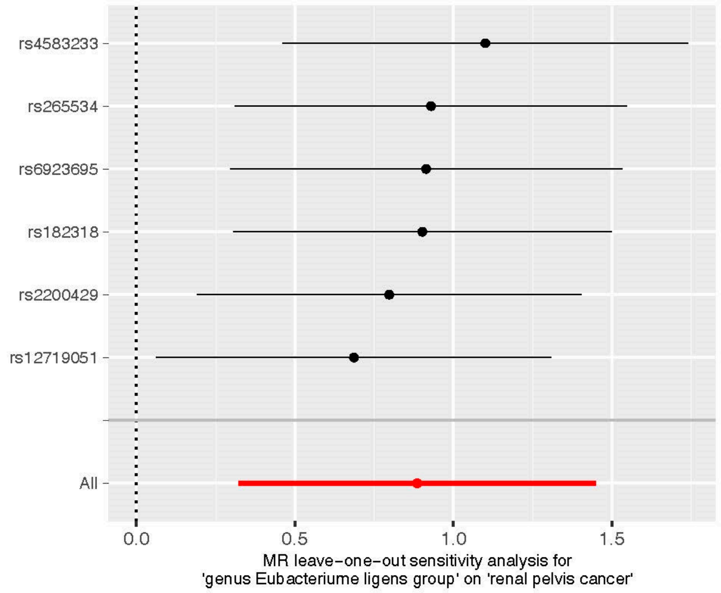

Figure S66. The leave-one-out test of genus Eubacterium eligens group on renal pelvis cancer

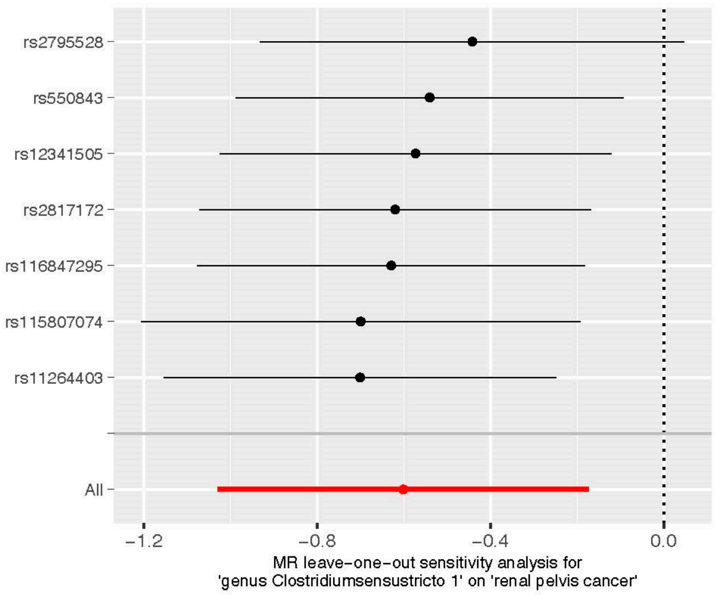

Figure S67. The leave-one-out test of genus Clostridiumsensustricto 1 on renal pelvis cancer

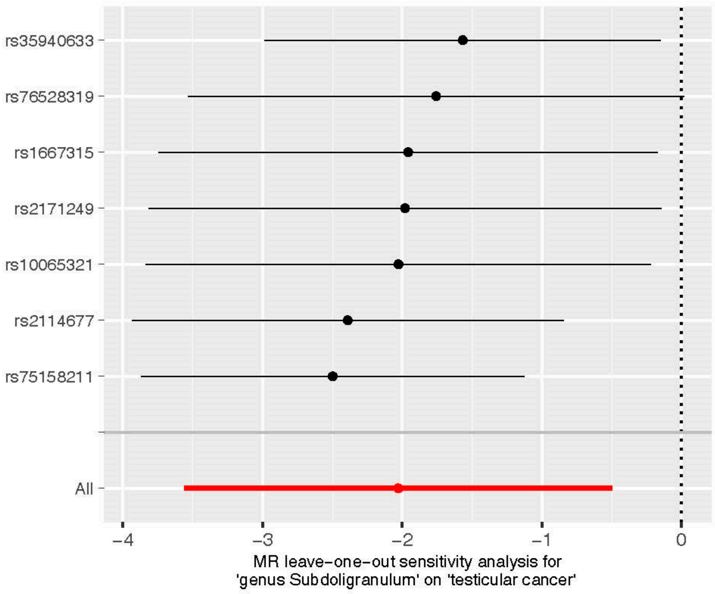

Figure S68. The leave-one-out test of genus Subdoligranulum on testicular cancer

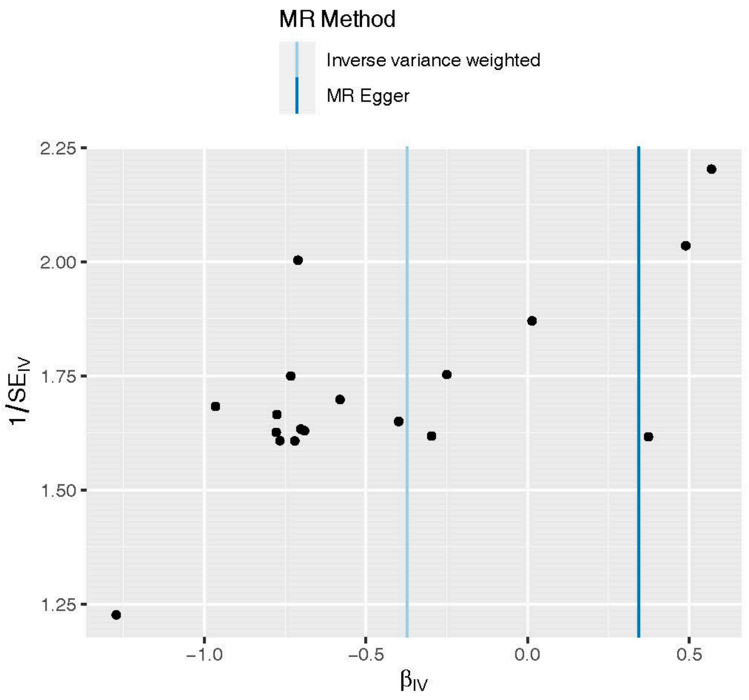

Figure S69. The funnel plot of family Rikenellaceae on bladder cancer

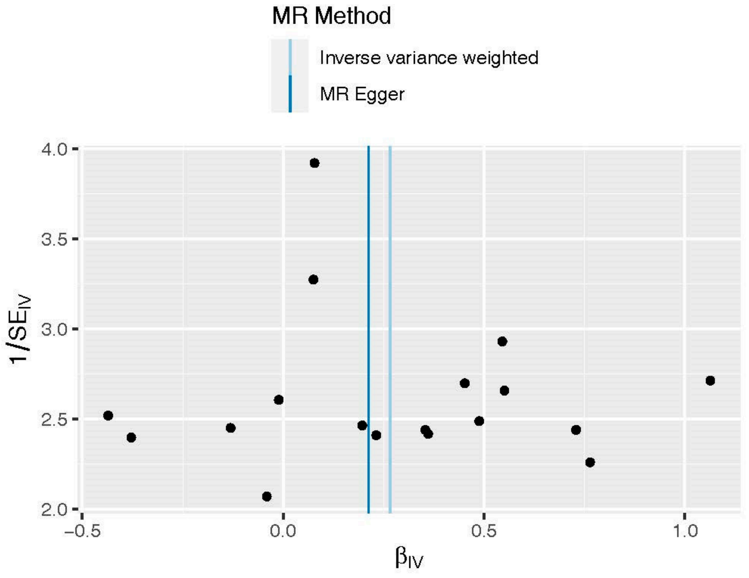

Figure S70. The funnel plot of genus Eubacterium ruminantium group on bladder cancer

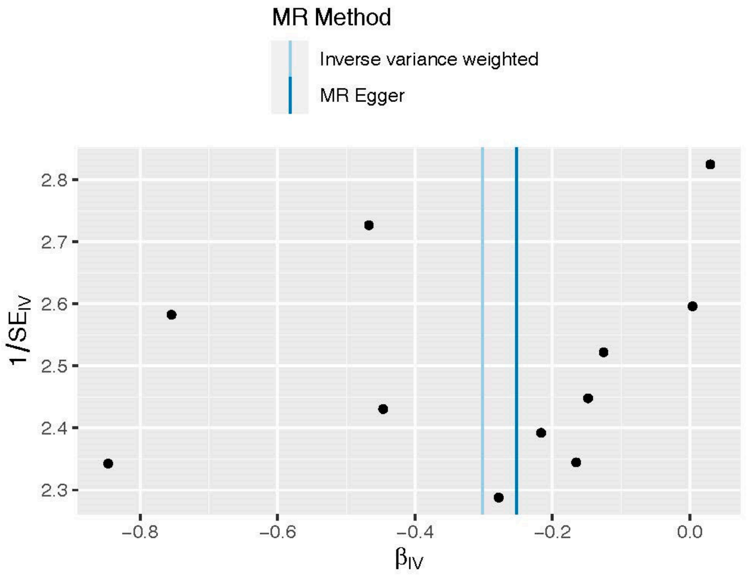

Figure S71. The funnel plot of genus Lachnospiraceae UCG001 on bladder cancer

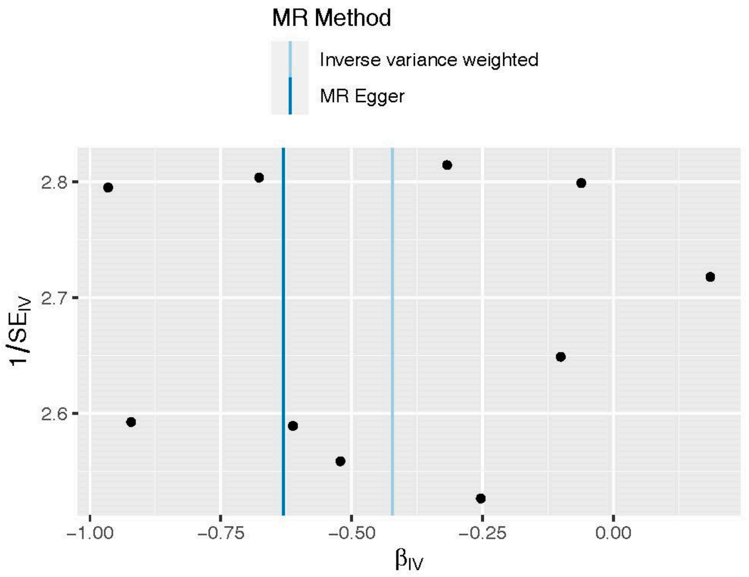

Figure S72. The funnel plot of genus Oscillibacte on bladder cancer

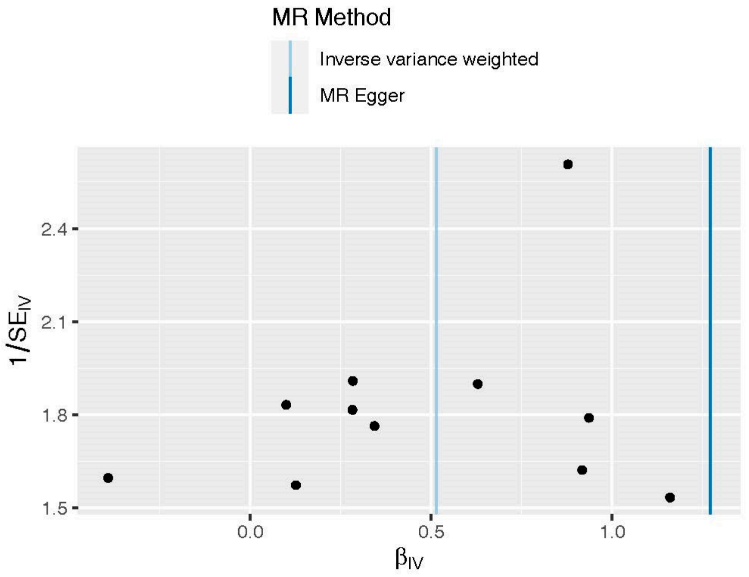

Figure S73. The funnel plot of genus Ruminococcaceae UCG013 on bladder cancer

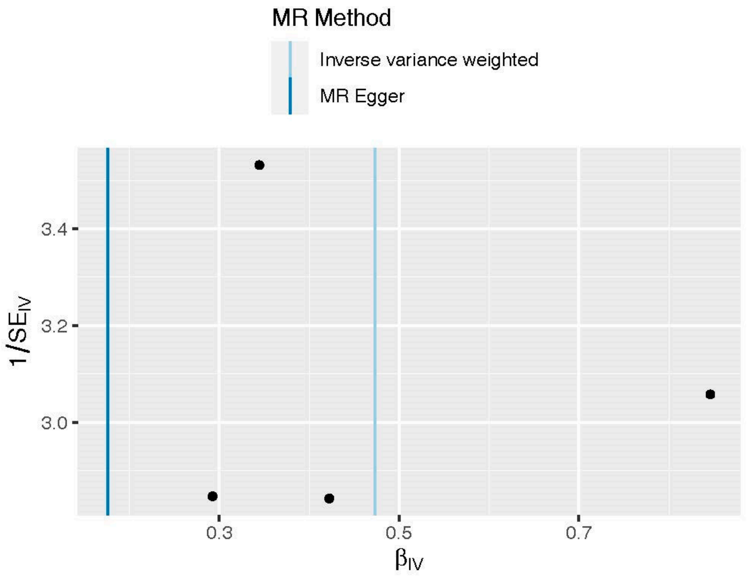

Figure S74. The funnel plot of genus Senegalimassilia on bladder cancer

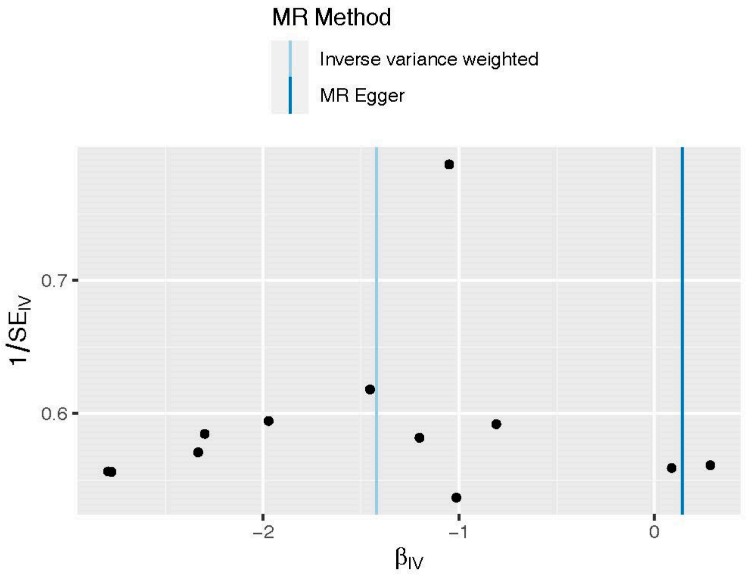

Figure S75. The funnel plot of genus Barnesiella on prostate cancer

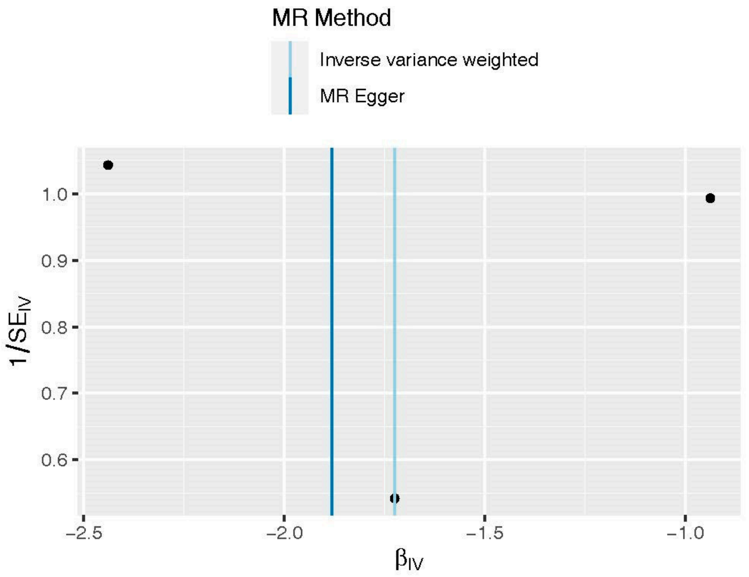

Figure S76. The funnel plot of genus Butyricicoccus on prostate cancer

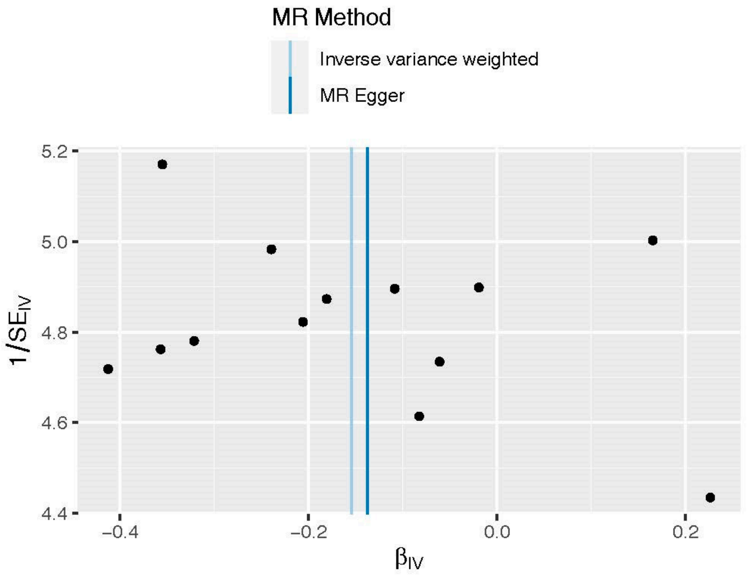

Figure S77. The funnel plot of genus Oscillibacter on prostate cancer

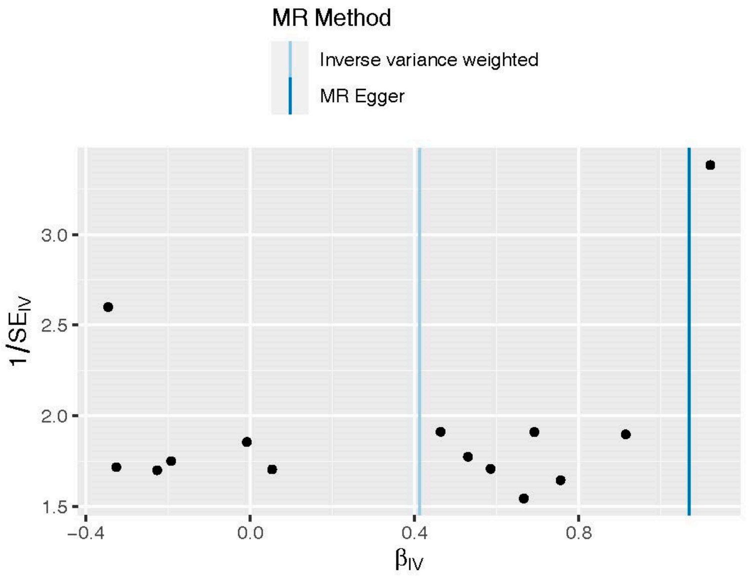

Figure S78. The funnel plot of genus Ruminococcaceae UCG005 on prostate cancer

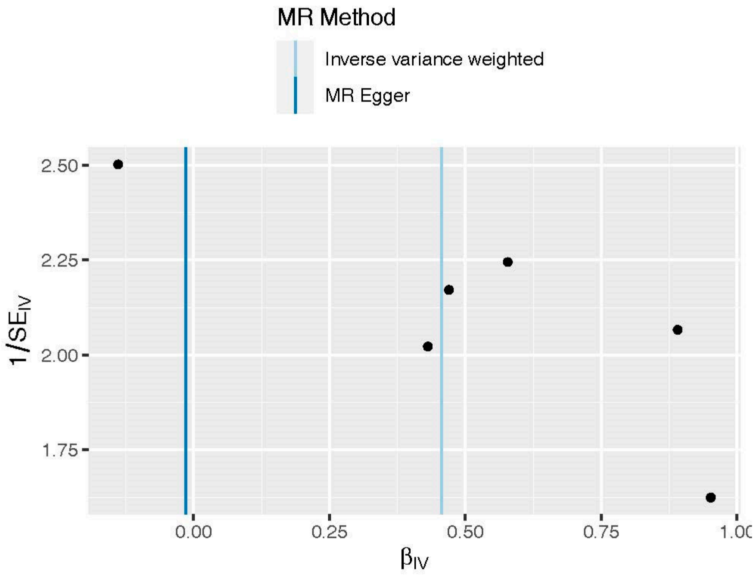

Figure S79. The funnel plot of class Alphaproteobacteria on renal cell cancer

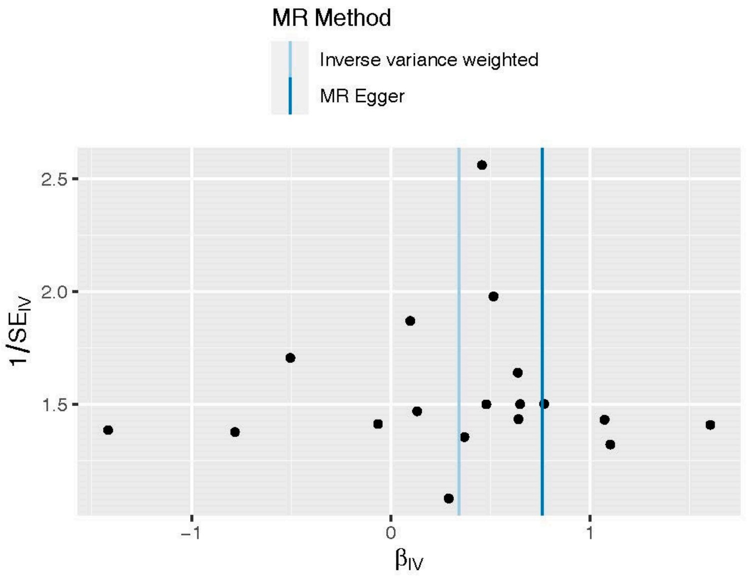

Figure S80. The funnel plot of class Bacilli on renal cell cancer

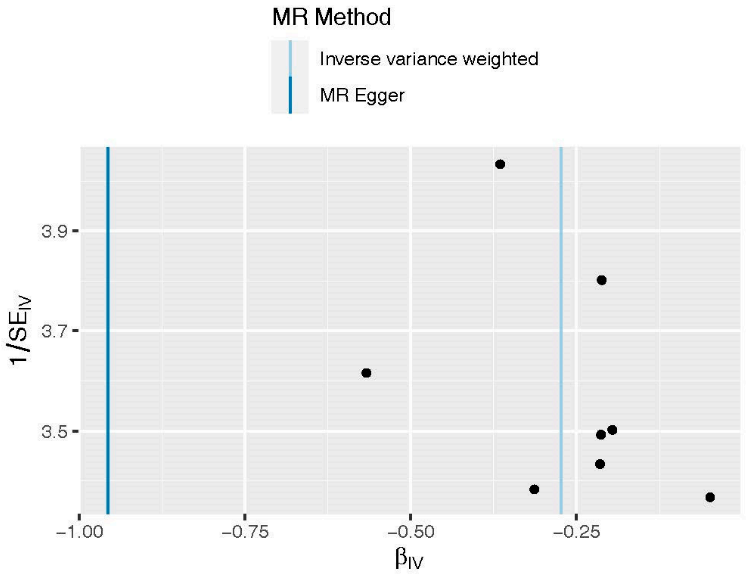

Figure S81. The funnel plot of family FamilyXI on renal cell cancer

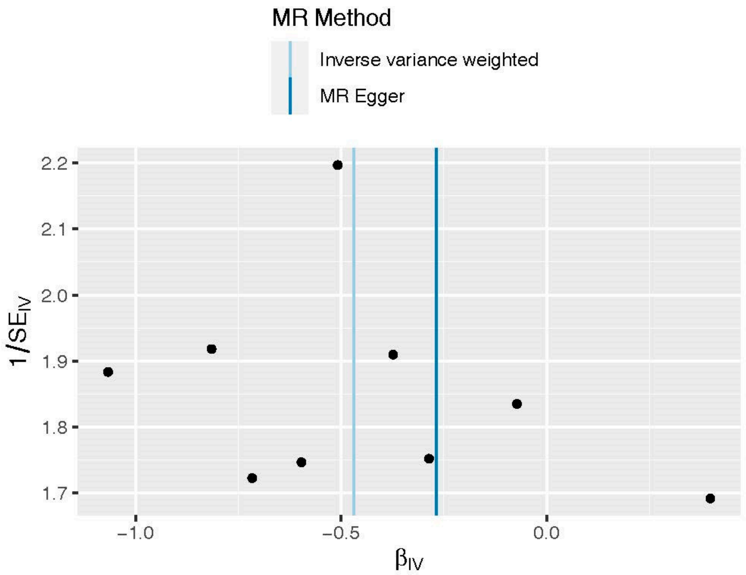

Figure S82. The funnel plot of genus Coprococcus 2 on renal cell cancer

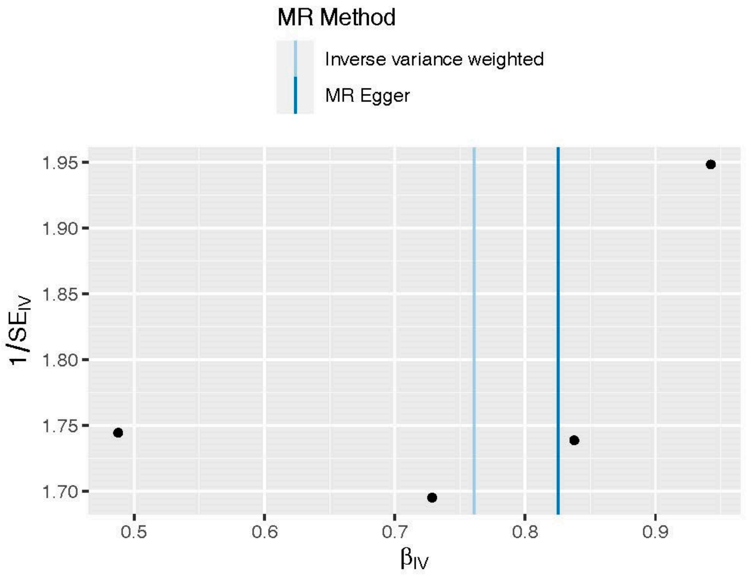

Figure S83. The funnel plot of genus Eubacterium brachy group on renal cell cancer

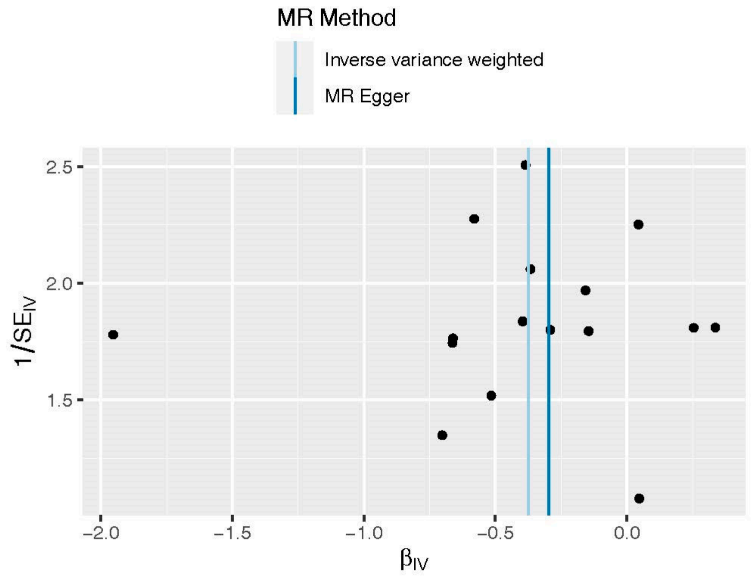

Figure S84. The funnel plot of genus Intestinimonas on renal cell cancer

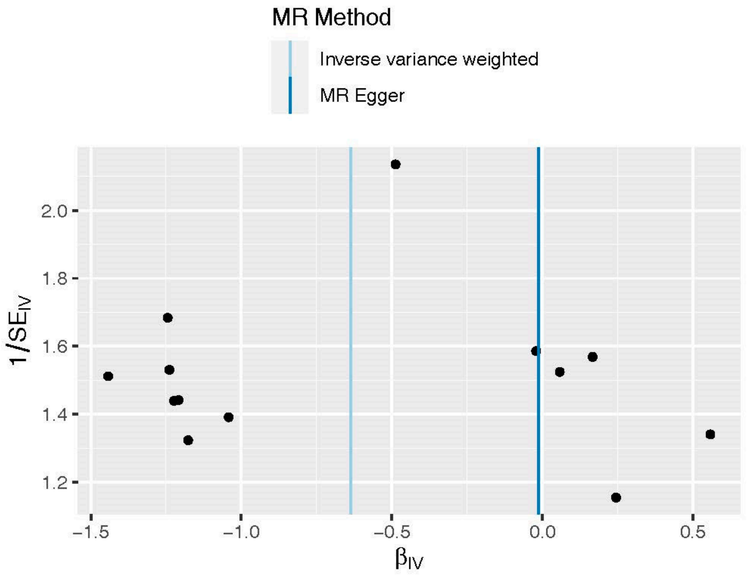

Figure S85. The funnel plot of genus Lachnoclostridium on renal cell cancer

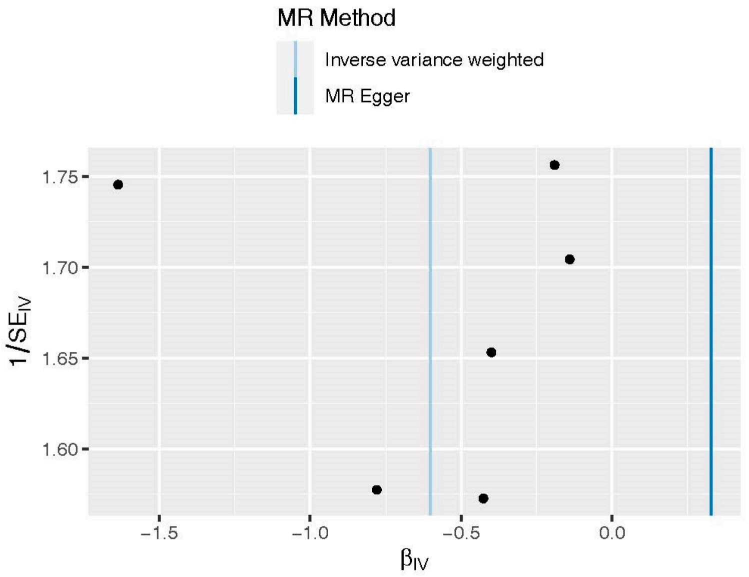

Figure S86. The funnel plot of genus Lactococcus on renal cell cancer

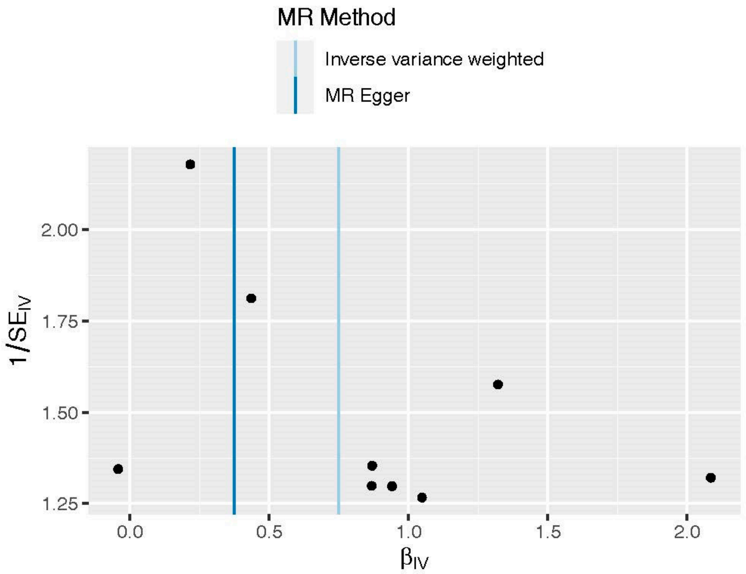

Figure S87. The funnel plot of family Christensenellaceae on renal cell cancer

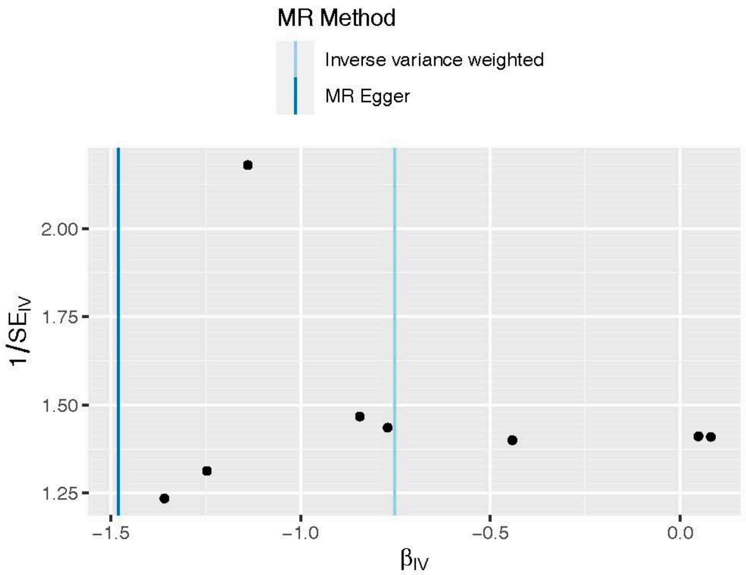

Figure S88. The funnel plot of family Clostridiaceae 1 on renal cell cancer

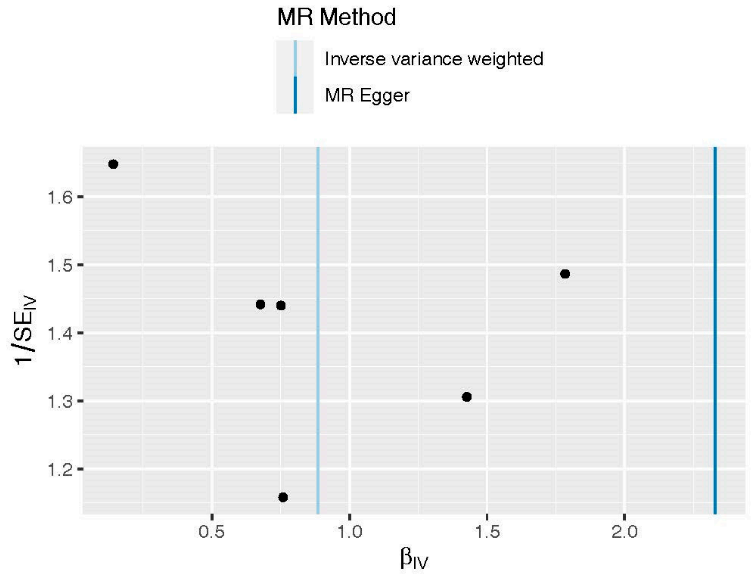

Figure S89. The funnel plot of genus Eubacterium eligens group on renal cell cancer

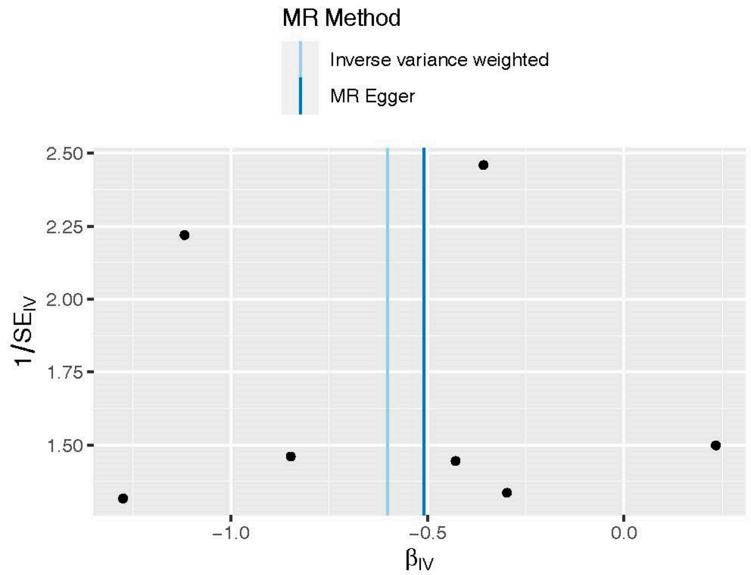

Figure S90. The funnel plot of genus Clostridiumsensustricto 1 on renal cell cancer

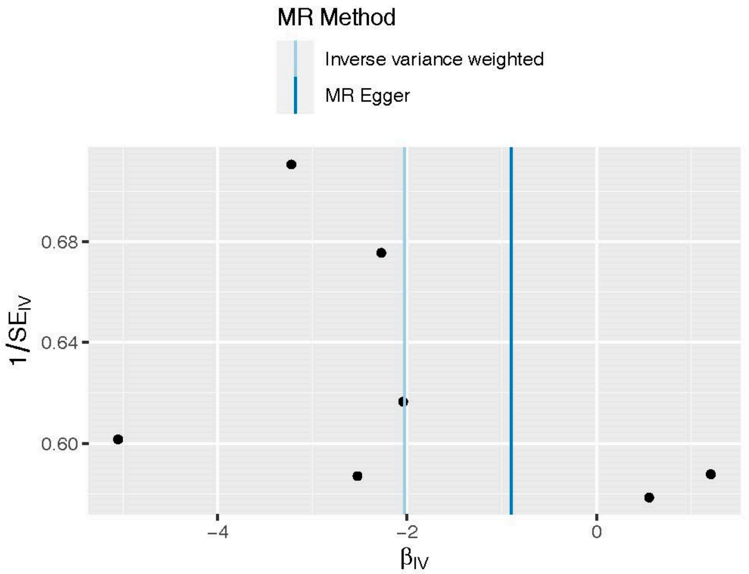

Figure S91. The funnel plot of genus Subdoligranulum on testicular cancer
